# DNA methylation changes facilitated evolution of genes derived from *Mutator*-like transposable elements

**DOI:** 10.1186/s13059-016-0954-8

**Published:** 2016-05-06

**Authors:** Jun Wang, Yeisoo Yu, Feng Tao, Jianwei Zhang, Dario Copetti, Dave Kudrna, Jayson Talag, Seunghee Lee, Rod A. Wing, Chuanzhu Fan

**Affiliations:** Department of Biological Sciences, Wayne State University, 5047 Gullen Mall, Detroit, MI 48202 USA; Arizona Genomics Institute, BIO5 Institute and School of Plant Sciences, University of Arizona, Tucson, AZ 85721 USA; T.T. Chang Genetics Resources Center, International Rice Research Institute, Los Baños, Laguna 4031 Philippines

**Keywords:** Comparative genomics, DNA methylation, GC content, Molecular evolution, MULEs, New genes, *Oryza*, Recombination rate

## Abstract

**Background:**

*Mutator*-like transposable elements, a class of DNA transposons, exist pervasively in both prokaryotic and eukaryotic genomes, with more than 10,000 copies identified in the rice genome. These elements can capture ectopic genomic sequences that lead to the formation of new gene structures. Here, based on whole-genome comparative analyses, we comprehensively investigated processes and mechanisms of the evolution of putative genes derived from *Mutator*-like transposable elements in ten *Oryza* species and the outgroup *Leersia perieri*, bridging ~20 million years of evolutionary history.

**Results:**

Our analysis identified thousands of putative genes in each of the *Oryza* species, a large proportion of which have evidence of expression and contain chimeric structures. Consistent with previous reports, we observe that the putative *Mutator*-like transposable element-derived genes are generally GC-rich and mainly derive from GC-rich parental sequences. Furthermore, we determine that *Mutator*-like transposable elements capture parental sequences preferentially from genomic regions with low methylation levels and high recombination rates. We explicitly show that methylation levels in the internal and terminated inverted repeat regions of these elements, which might be directed by the 24-nucleotide small RNA-mediated pathway, are different and change dynamically over evolutionary time. Lastly, we demonstrate that putative genes derived from *Mutator*-like transposable elements tend to be expressed in mature pollen, which have undergone de-methylation programming, thereby providing a permissive expression environment for newly formed/transposable element-derived genes.

**Conclusions:**

Our results suggest that DNA methylation may be a primary mechanism to facilitate the origination, survival, and regulation of genes derived from *Mutator*-like transposable elements, thus contributing to the evolution of gene innovation and novelty in plant genomes.

**Electronic supplementary material:**

The online version of this article (doi:10.1186/s13059-016-0954-8) contains supplementary material, which is available to authorized users.

## Background

*Mutators* are class II DNA transposable elements (TEs) and have propagated widely across both prokaryotic and eukaryotic genomes through a “cut-and-paste” mechanism. The *Mutator* system was first reported in maize [1, 2] and was later found in other plants, bacteria, fungi and protozoans [3–6]. *Mutator*-like transposable elements (MULEs) are especially pervasive in higher plant genomes such as rice (*Oryza sativa*), in which more than 10,000 copies have been identified [6–9]. The typical structure of a MULE includes terminated inverted repeats (TIRs; usually 100–500 bp) flanking an internal sequence and one target site duplication (TSD; usually 8–11 bp) flanking each TIR [7, 10]. MULEs can be classified into two categories based on the properties of their internal sequences: (1) autonomous MULEs, containing internal sequences that encode transposases; and (2) non-autonomous MULEs, lacking the transposase gene. The transposase encoded by autonomous MULEs can transpose both autonomous and non-autonomous MULEs [11, 12]. Studies have demonstrated that MULEs can play important roles in the generation of potentially functional genes and in modulating genic GC-content distribution in monocot genomes [7, 8, 13, 14].

New genes can be created through various mechanisms, such as whole-genome duplication, small-scale duplication, illegitimate recombination, horizontal gene transfer, gene fusion, de novo origination from non-coding DNA sequence, RNA mediated retrotransposition, and dispersion/origination through TEs [13, 15–22]. It has been demonstrated that non-autonomous MULEs can capture ectopic genomic sequences, such as gene fragments, and transpose them into new genomic locations, thereby forming putative new gene structures [8, 23, 24].

The discovery that MULEs can capture gene fragments was first reported in maize [23]. More recently, genome-wide analyses and individual case studies have revealed that non-autonomous MULEs carrying intact or partial gene fragments (termed Pack-MULEs) are abundant in many plant genomes [7, 10]. For example, analysis of the gold standard rice (i.e., *Oryza sativa* ssp. *japonica*) reference genome revealed the presence of more than 3000 Pack-MULEs [7, 8]. Analyses of the internal sequences of Pack-MULEs have shown that they have the potential to serve as functional genes based on transcription (i.e., mRNA and small RNA), translation, and selective constraint evidence [8, 23]. Theoretic models of how MULEs acquire new sequences propose that internal sequences and new TIR regions are introduced into MULEs by DNA repair and conversion of gaps on stem-loop structures or the invasion of excision regions of MULEs into ectopic sequences [7, 25, 26].

Due to the high abundance of MULEs and their remarkable functional roles in genome evolution, it is imperative to elucidate the origination, evolutionary processes, and regulatory mechanisms of MULE-derived genes in plant genomes. Further, answers to these questions could shed light on the evolutionary processes and fates of TE-derived genes in general. To address these questions, comparative genomic and phylogenetic analyses based on a set of high-quality genomic data from closely related species are required. In this study we interrogated a recently released set of genomes and transcriptomes from ten *Oryza* species (*O. sativa* ssp*. japonica*, *O. sativa* ssp*. indica*, *O. nivara*, *O. rufipogon*, *O. barthii*, *O. glaberrima*, *O. glumaepatula*, *O. meridionalis*, *O. brachyantha*, *and O. punctata*) and one outgroup species, *Leersia perrieri*, for MULE-derived putative genes. We systematically profiled the formation of these MULE-derived putative genes at both the genus and species level and determined the origination mechanisms and evolutionary processes leading to their origination. Our results suggest that DNA methylation may be one of the primary mechanisms modulating the evolution of MULE-derived genes in plant genomes.

## Results

### Identification of non-autonomous MULEs across an 11-genome dataset

**Fig. 1:**
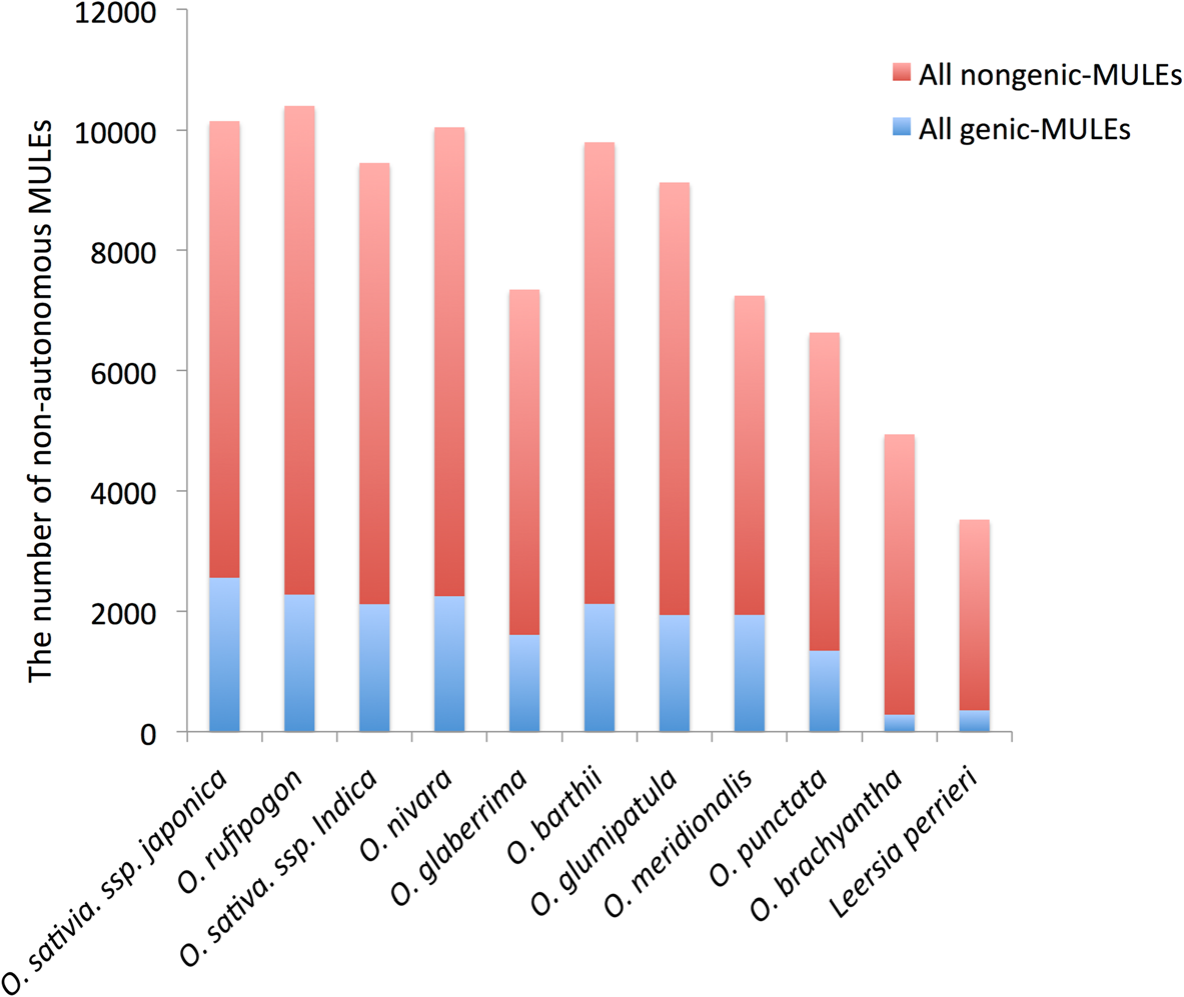
Number of non-autonomous MULEs across the 11-genome dataset. Non-autonomous MULEs were divided into two categories, genic-MULEs and nongenic-MULEs. The proportion of genic-MULEs among non-autonomous MULEs is shown in *blue* and the proportion of nongenic-MULEs is shown in *red*

**Fig. 2:**
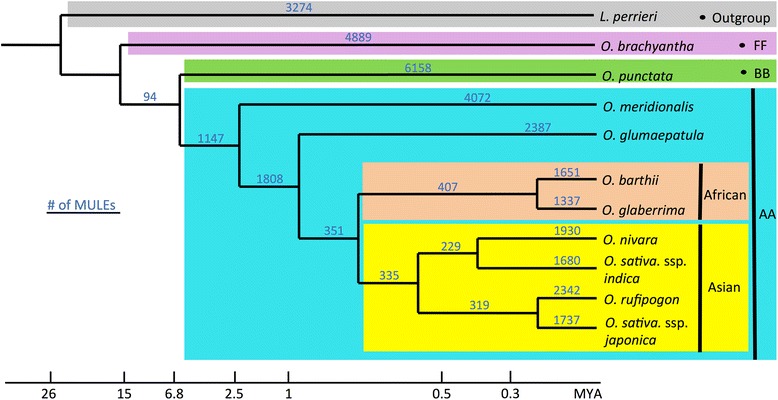
Number of non-autonomous MULEs across the 11-genome dataset. Based on the presence and absence of non-autonomous MULEs in the phylogenetic tree of ten *Oryza* and one *Leersia* species and the evolutionary parsimony principle, we inferred the number of non-autonomous MULEs for each external species and internal branch across the ten *Oryza* and one *Leersia* phylogenetic tree

To understand the dynamics of MULE origination and evolution across a ~20 million year time span within a single genus, we first identified the majority of non-autonomous MULEs for each of 11 genome assemblies (see “Methods”). Overall, between ~7000 and 10,000 elements were detected in each AA and BB genome assembly, which is similar to the number of MULEs previously identified in the *O. sativa* ssp*. japonica* RefSeq [7], and ~4000 and 5000 in the basal *Oryza* species *O. brachyantha* and the outgroup *L. perrieri* (Fig. 1). Based on homolog searches, local syntenic region comparisons, and the phylogeny of the ten *Oryza* and one *Leersia* species (see “Methods” for more details), we further defined the presence and absence of each non-autonomous MULE in the 11 species and internal lineages. Using this information, combined with the principle of evolutionary parsimony [27], we inferred the evolutionary divergence times, namely the approximate age, of all annotated non-autonomous MULEs (Fig. 2), which allowed for the identification of species-specific MULES across the *Oryza* genus. As a result, we identified ~1000–2000 species-specific MULEs in domesticated *Oryza* species, including *O. sativa* and *O. glaberrima*, and their wild progenitors. Around 2000–6000 species-specific MULEs were found in the basal *Oryza* species, including O. *glumipatula*, *O. meridionalis*, *O. punctata*, and *O. brachyantha*, and the outgroup species *L. perrieri*. We also found that fewer MULEs were present in the internal branches, which were referred as the ancestors of multiple *Oryza* species (Fig. 2).

To validate the age of the non-autonomous MULEs estimated by the above phylogenetic approach and evolutionary parsimony principle, we further inferred the amplification time of each non-autonomous MULE by analyzing the sequence divergence of a MULE and its most similar paralogous non-autonomous MULE that belongs to the same MULE TIR family. Using an all-by-all BLAT search of all non-autonomous MULEs for each species, we identified the most similar element with the same MULE TIR for each MULE. These paralogous MULEs most likely were derived from each other or share a common ancestor (i.e., the most recent common ancestor) and thus their sequence divergence since formation can be used to infer their amplification time [28]. Hence, we aligned sequences of those paralogous MULEs and estimated their sequence divergence using the baseml module from PAML to calculate their amplification times.

We categorized MULEs based on their origination time points inferred from the presence and absence of MULEs in the *Oryza* phylogenetic species tree and drew density distributions of the amplification times of MULEs for each origination time point category for the 11 species. As shown in Additional file 1: Figure S1, MULEs in internal branches have longer amplification times compared with the MULEs in the terminal nodes of the *Oryza* phylogenetic tree. These results demonstrate that the origination time of MULEs inferred from the presence and absence of MULEs in the phylogeny tree is consistent with the amplification time computed from the sequence divergence of paralogous MULEs for each species.

### Identification of open reading frames derived from non-autonomous MULEs

Non-autonomous MULEs have been shown to transpose ectopic genomic sequences to new genomic locations and potentially form novel functional gene structures [5, 9, 23, 24, 29, 30]. Based on both MAKER and GlimmerHMM annotations of all 11 genome assemblies, we searched for the presence of intact open reading frames (ORFs) located within all identified non-autonomous MULEs and classified them into two groups: (1) genic-MULEs, the ones that have overlap with annotated and intact transcripts (see “Methods”); and (2) nongenic-MULEs, the ones that do not meet the criteria for genic-MULEs. Both genic-MULEs and the previously defined Pack-MULEs are non-autonomous MULEs that do not contain transposase fragments. Pack-MULEs are defined to carry non-hypothetical parental protein fragments while genic-MULEs are merely required to contain ORFs. Since new genes may originate from MULE sequences without well-defined protein structures or sequences too old to be identified by sequence homology, the genic-MULE dataset developed in this study is ideal to study the origination and evolution of MULE-derived genes. Analysis of this dataset revealed the presence of between ~1000 and 2500 genic-MULEs (i.e., ~20–25 % of non-autonomous MULEs) for most *Oryza* species, with the exception of the basal *O. brachyantha* and *L. perrieri* species, which contained about 300 elements each (i.e., 6–10 % of non-autonomous MULEs) (Fig. 1).

We defined MULE-derived putative genes as those in which the annotated ORFs contained at least 150 bp of coding sequence (CDS; i.e., encode 50 amino acids or more), the start and stop codons were intact, and at least 30 % of their transcript lengths have overlap with non-autonomous MULEs. Overall, we identified ~2000–3000 MULE-derived putative genes in the AA and BB genome *Oryza* species and ~350–500 MULE-derived putative genes in *O. brachyantha* and *L. perrieri* (Table 1). Among them, ~2 % appear to be species-specific and originated from species-specific MULEs in domesticated *Oryza* species and their wild progenitors (Table 1). Based on the number of species-specific MULE-derived putative genes and the divergence times of the *Oryza* species, we infer that the rate of the new gene origination via non-autonomous MULEs is in the order of 14 to 222 putative genes per million years per genome (Additional file 2: Table S1).

**Table 1:** Number of MULE-derived putative genes identified across the 11-genome data set

Species	Number of all MULE-derived putative genes	Number of species-specific MULE-derived putative genes
*O. sativa* ssp. *japonica*	3245	49
*O. sativa* ssp. *indica*	2645	48
*O. rufipogon*	2792	39
*O. nivara*	2794	28
*O. glaberrima*	1996	23
*O. barthii*	2623	22
*O. glumipatula*	2429	106
*O. meridionalis*	2468	537
*O. punctata*	1823	1293
*O. brachyantha*	354	238
*Leersia perrieri*	477	368

### Structure, transcription, and functional constraints of MULE-derived genes

MULE-derived putative genes tend to have simple exon–intron structures with around half of them containing a single exon and fewer with multiple exons (Table 2). The parental genes of MULE-derived putative genes are the genes from which MULEs capture their internal sequences. Based on sequence homology searches, we attempted to identify the parental genes of as many MULE-derived putative genes as possible in all 11 species (see “Methods”). For most *Oryza* species, we found that ~30–40 % of the MULE-derived putative genes acquired their ORF sequences from at least one parental gene. Interestingly, ~100–300 MULE-derived putative genes (5–10 %) acquired their ORF sequences from at least two parental genes, thereby forming novel chimeric gene structures (Table 2).

**Table 2:** Structure, transcription, and functional constraint values of MULE-derived putative genes across the 11-genome dataset

Species	Parental ≥1	Parental ≥2	Exon = 1	Exon ≥2	FPKM > 0	Ka/Ks < 1*	Ka/Ks > 1*
*O. sativa* ssp*. japonica*	1186	264	1745	1500	628	89	3
*O. sativa* ssp*. indica*	922	187	1412	1233	N/A	76	3
*O. rufipogon*	1042	258	1523	1269	563	99	4
*O. nivara*	1161	285	1448	1346	688	72	1
*O. glaberrima*	687	130	1118	878	662	45	1
*O. barthii*	743	115	1485	1138	465	38	2
*O. glumipatula*	939	247	1278	1151	702	44	1
*O. meridionalis*	916	262	1408	1060	754	84	2
*O. punctata*	393	34	1106	717	202	28	3
*O. brachyantha*	123	27	185	169	146	7	0
*Leersia perrieri*	78	13	306	171	184	1	0

*The q value of the likelihood ratio test of Ka/Ks ratio ≤0.05

To determine whether any of the MULE-derived putative genes in our 11-genome data set are under functional constraints and to detect selective forces after MULE acquisition [28], we estimated Ka/Ks values based on sequence divergence of MULE-derived putative genes and the most similar paralogous non-autonomous MULEs using a modified gKaKs pipeline with Codeml option from PAML [31, 32]. For most *Oryza* species, ~100 MULE-derived putative genes (i.e., ~4 % of the total number of putative genes detected) had Ka/Ks values significantly less than 1 (likelihood ratio test, false discovery rate q value <0.05; Table 2). And a few putative genes (0–4) had Ka/Ks values significantly larger than 1 (likelihood ratio test, false discovery rate q value <0.05; Table 2).

To determine the number of MULE-derived putative genes that are transcribed, we analyzed baseline RNA-seq data derived from panicle, root, and leaf tissues from 10 of the 11 species. We mapped all available RNA-seq data to the MULE-derived putative gene data set and measured gene expression intensity by computing fragments per kilobase of exon per million reads (FPKM) values (see “Methods”). By calibration with expression profiles from intergenic sequences, we considered a FPKM value >0 as the cutoff threshold for evidence of expression. Overall, about 20–40 % of the MULE-derived putative genes in most species had FPKM values >0 in at least one tissue (Table 2).

### GC-rich MULE-derived genes from GC-rich parental sequences

**Fig. 3:**
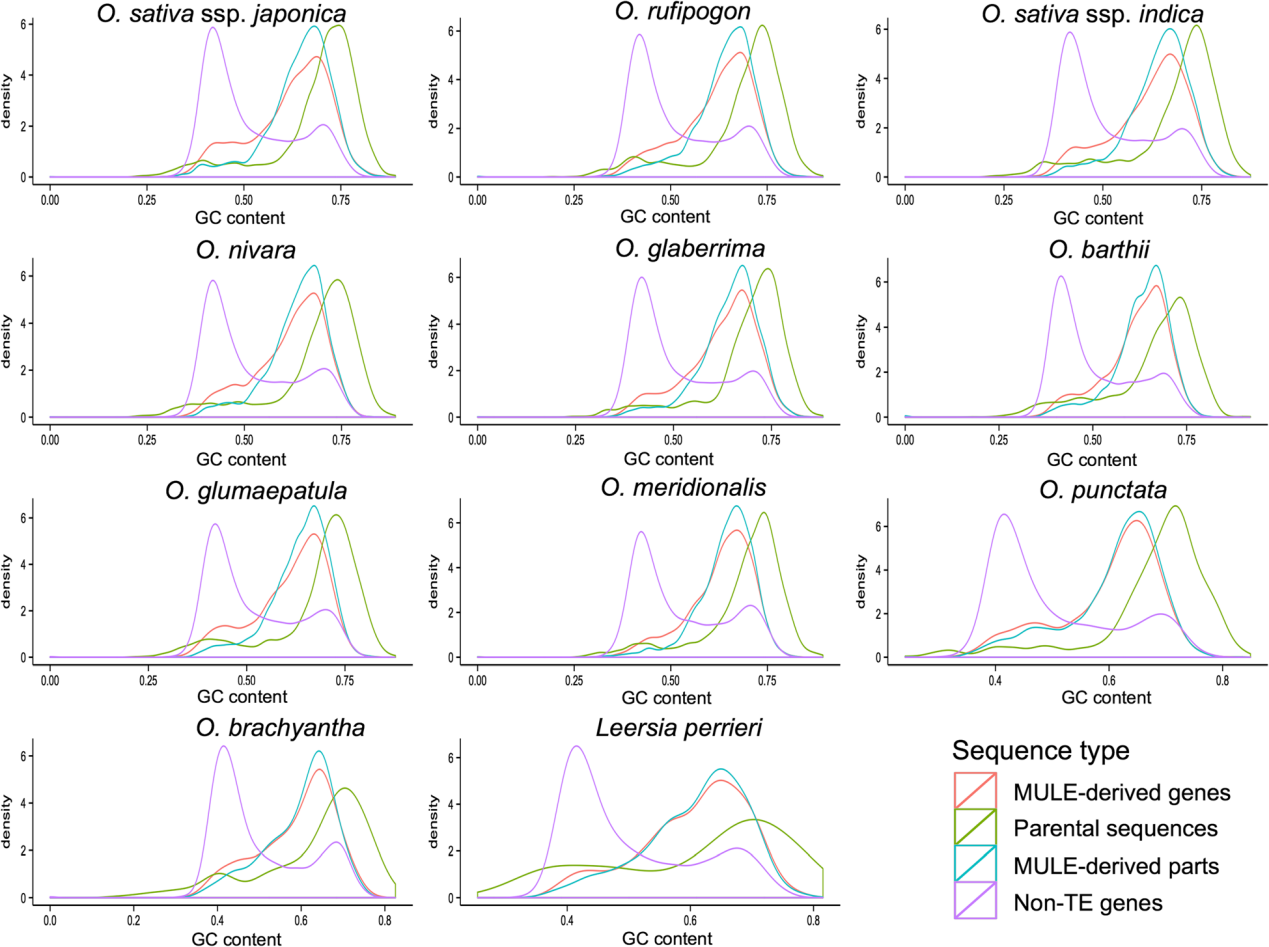
GC-content distributions of non-TE genes, MULE-derived putative genes, MULE internal sequences of MULE-derived putative genes, and the parental sequences of MULE-derived putative genes. Density distribution of GC content of non-TE genes (in *purple*), MULE-derived putative genes (in *red*), MULE internal sequences of MULE-derived putative genes (in *blue*), and the parental sequences of MULE-derived putative genes (in *green*). MULE-derived putative genes, especially the parts derived from MULEs, have a much higher GC content than non-TE genes across the 11 genome dataset (Wilcoxon rank sum test, *P* value < 2.2e-16). The GC content of MULE-derived putative genes is less than that of the MULE internal sequences of MULE-derived putative genes which is less than that of the parental sequences of MULE-derived putative genes (Wilcoxon rank sum test, *P* < 0.05, except for the comparison of MULE-derived putative genes and MULE internal sequences in *L. perrieri* and *O. brachyantha*)

Previous studies showed that MULEs selectively capture ectopic GC-rich sequences and insert them into the 5′ end of gene ends, thereby modulating the GC gradient of monocot genes [7, 8]. To test this observation, we calculated the GC content, i.e., the proportion of GC bases within the sequence, of MULE-derived putative genes. We consistently found that *Oryza*/*Leersia* MULE-derived putative genes, especially the gene fragments derived from MULEs, have a much higher GC content compared with non-TE genes across the 11 genome dataset (Fig. 3; Wilcoxon rank sum test, *P* value <2.2e-16). Further, the GC content of their parental sequences is even higher, suggesting that MULEs acquired parental sequences selectively (Fig. 3; GC content of MULE-derived putative genes is less than that of the MULE internal sequences of MULE-derived putative genes which is less than that of the parental sequences of MULE-derived putative genes; Wilcoxon rank sum test, *P* < 0.05, except for the comparison of MULE-derived putative genes and the MULE internal sequences in *L. perrieri* and *O. brachyantha*). Moreover, we examined the parental sequences of species-specific MULE-derived putative genes which originated after the divergence of each species and likely represent the most recent sequence capture events and found that they also are GC-rich, suggesting that MULEs prefer to capture GC-rich sequences initially.

### Parental sequences of MULE-derived putative genes are located in regions of the genome that are hypomethylated and highly recombinogenic

As previously proposed, MULEs acquire parental sequences by using DNA repair/conversion mechanisms through invasion into ectopic sequences [26]. This motivated us to investigate whether the chromatin structure (i.e., methylation status, recombination rate) of MULE parental sequences has special signatures that make them more susceptible for invasion. As recombination rates are positively associated with chromatin remodeling [33], they may be related to parental sequence captured by MULEs. To address this hypothesis, we analyzed the recombination rate of the parental sequences of MULE-derived putative genes in *O. sativa* ssp. *japonica* (see “Methods” for more details). Our analysis showed that these parental sequences are primarily located in regions that have significantly higher recombination rates than non-TE genes, which served as controls (Wilcoxon rank sum test, *P* = 1.874e-09/3.755e-08).

**Fig. 4:**
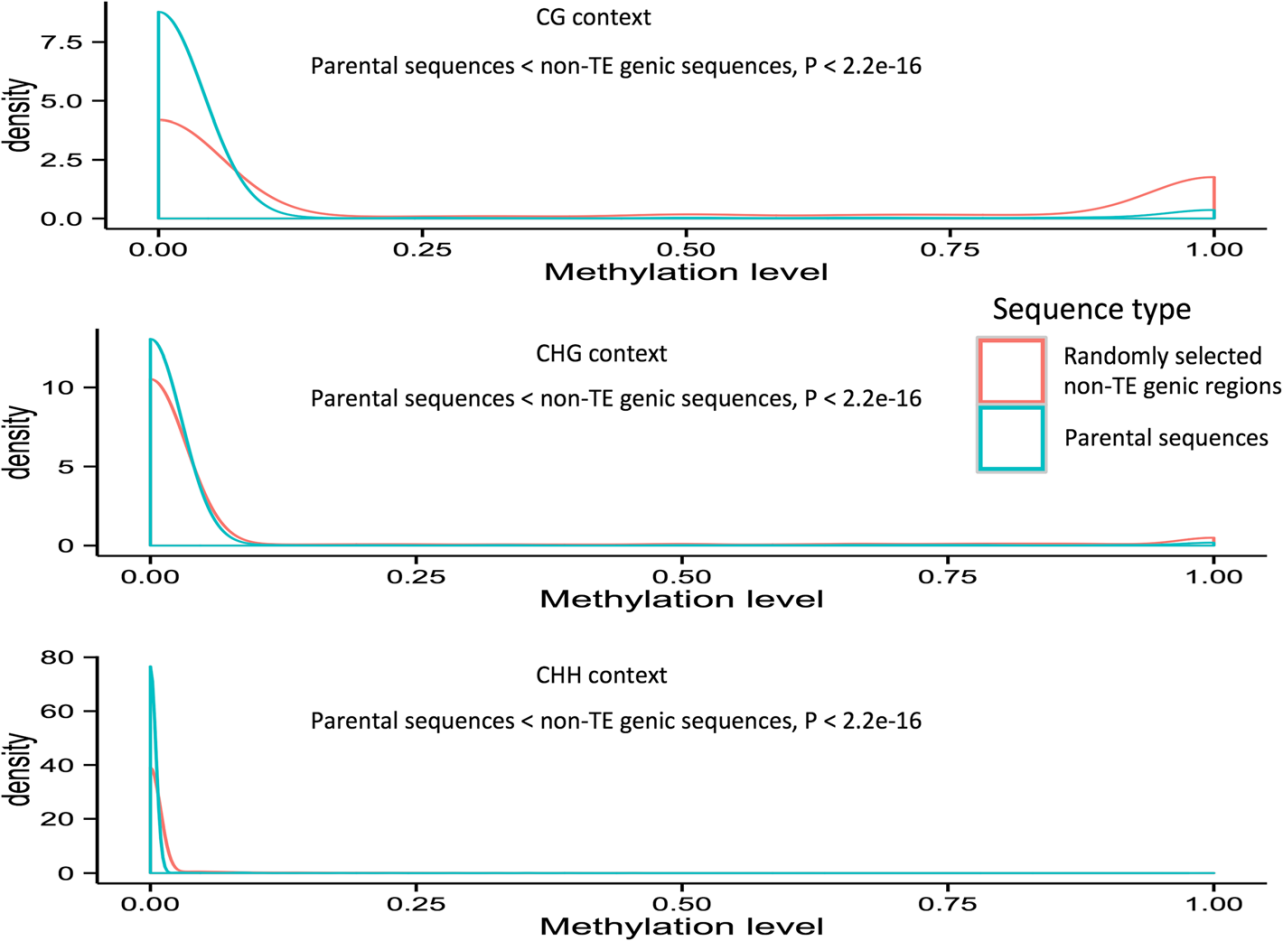
Methylation level distribution of parental sequences of MULE-derived putative genes and randomly selected non-TE genic regions across the *O. sativa* ssp. *japonica* genome. A density distribution of the methylation levels of parental sequences of MULE-derived putative genes (*green*) and that of randomly selected non-TE genic regions across the *O. sativa* ssp. *japonica* genome (*red*) in three cytosine contexts. The figure shows that the parental sequences of MULE-derived putative genes have lower methylation levels compared with non-TE genic regions

Since high DNA methylation levels are generally associated with condensed chromatin structure [34], we also wanted to test whether DNA methylation levels of the parental sequences of MULE-derived putative genes are lower than those of control sequences. Using a DNA methylation dataset for *O. sativa* ssp*. japonica* (see “Methods”), we cataloged the methylation status of all parental sequences where DNA methylation data were available (~1742) and compared these data with randomly selected genic regions (~12,064) from a set of control non-TE genes across the rice genome. Indeed, we observed that the DNA methylation levels of the parental sequences of MULE-derived putative genes were low, with the majority around zero, which was significantly lower than the non-TE gene controls (Wilcoxon rank sum test, *P* < 2.2e-16 in CG, CHG, and CHH contexts; Fig. 4). We also analyzed an additional DNA methylation dataset from the *O. nivara* genome and found a similar trend (Additional file 1: Figure S2).

### Dynamic methylation changes and evolution of genic-MULEs

**Fig. 5:**
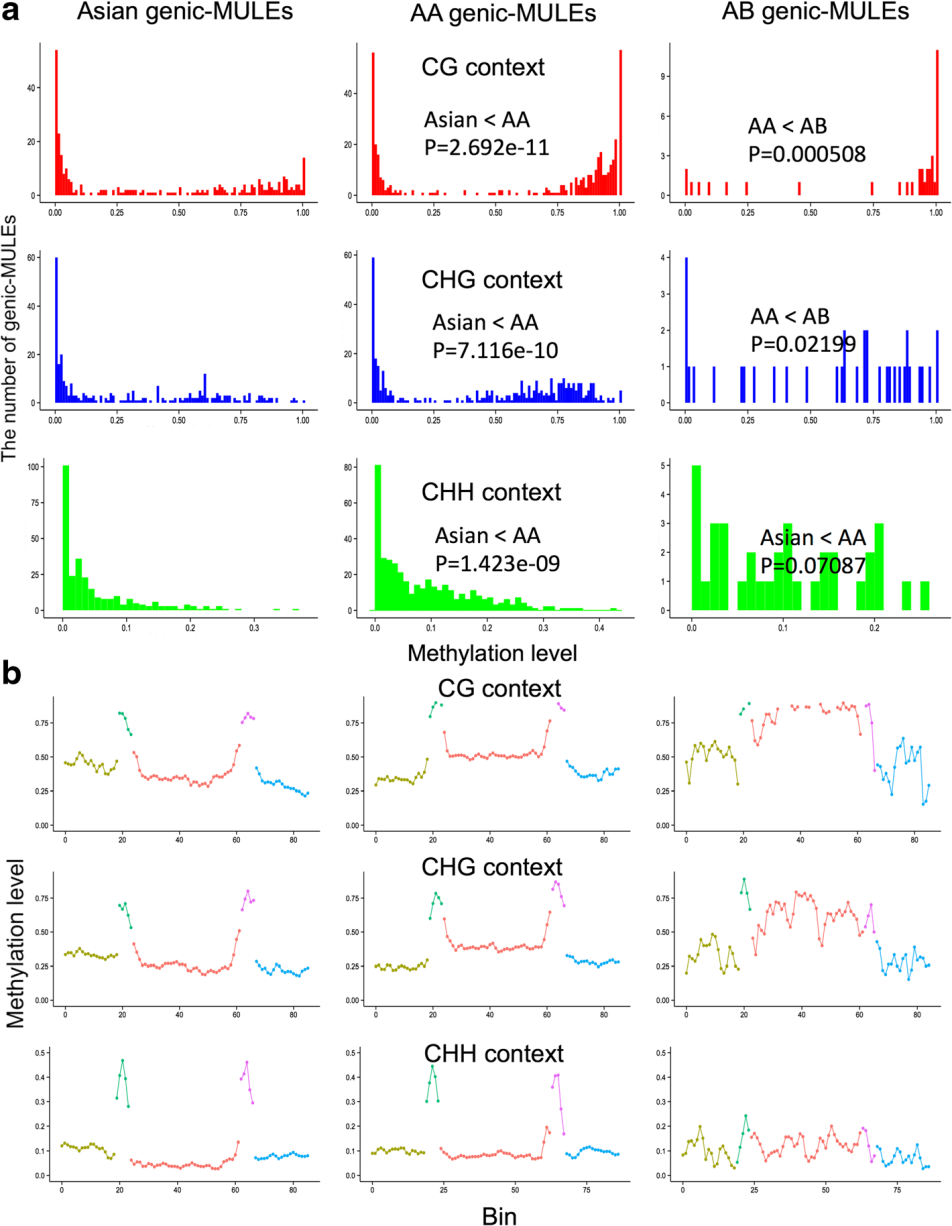
Methylation levels of genic-MULEs identified in the *O. sativa* ssp. *japonica* genome change over evolutionary time. **a** The methylation levels of MULE internal sequences with three evolutionary ages (Asian genic-MULEs, AA genic-MULEs, and AB genic-MULEs) in three cytosine contexts (CG context in *red*, CHG context in *blue*, and CHH context in *green*). Methylation levels of MULE internal sequences increase over time in three cytosine contexts (Wilcoxon rank sum test, *P* < 0.05, except the comparison of AA genic-MULEs and AB genic-MULEs in the CHH context, *P* = 0.07087). **b** A sliding window analysis of the average methylation level in 500-bp upstream flanking sequences (*yellow*), left TIR (*green*), internal sequence (*red*), right TIR (*purple*), and 500-bp downstream flanking sequences (*blue*) of genic-MULEs with three evolutionary ages in three cytosine contexts. Methylation levels vary across the different regions of MULEs. Methylation levels of the TIR regions in the CHH context decrease over evolutionary time (Wilcoxon rank sum test, *P* < 0.0501)

Since the parental sequences of MULE-derived putative genes are undermethylated relative to other non-TE genic sequences, we next determined the methylation patterns of genic-MULEs themselves and whether their methylation patterns change over evolutionary time. To perform this analysis we chose three groups of genic-MULEs in the *O. sativa* ssp*. japonica* genome with increasing evolutionary divergence times based on our previously constructed phylogenetic tree of the genic-MULEs: (1) “Asian genic-MULEs” originating within Asian *Oryza* species less than 0.8 million years ago (MYA); (2) “AA genic-MULEs” originating after the AA genome *Oryza* split from the BB genome species but before the AA genome species diverged ~2.5–6.8 MYA; and (3) “AB genic-MULEs” originating before the AA and BB genome species split ~6.8–26 MYA (Fig. 2). Overall, the methylation levels of genic-MULE internal sequences in all three cytosine contexts were found to increase over evolutionary time as the methylation levels in the internal regions of Asian genic-MULEs are less than those of AA genic-MULEs which are less than those of AB genic-MULEs (Wilcoxon rank sum test, *P* < 0.05, except the comparison of AA genic-MULEs and AB genic-MULEs in the CHH context *P* = 0.07087; Fig. 5a).

Further, gene body methylation levels of MULE-derived putative genes also increased as the associated genic-MULEs became older, i.e., the methylation levels in the gene bodies of Asian MULE-derived putative genes were less than those of AA MULE-derived putative genes which were less than those of AB MULE-derived putative genes (Wilcoxon rank sum test, *P* < 0.05, except the comparison of AA MULE-derived putative genes and AB MULE-derived putative genes in the CHH context; Additional file 1: Figure S3a). We further observed that the methylation levels of the TIR regions in the CHH context decrease over time following a pattern where methylation levels in the TIRs of Asian genic-MULEs are greater than those of AA genic-MULEs which are greater than those of AB genic-MULEs (Wilcoxon rank sum test, *P* < 0.0501; Fig. 5b).

Finally, the methylation levels in promoters of MULE-derived putative genes are similar to those in the mixed patterns of MULE TIR and internal regions, with methylation levels in the promoters of Asian MULE-derived putative genes less than those of AA MULE-derived putative genes for CG, CHG, and CHH contexts, and those of AA MULE-derived putative genes are greater than those of AB MULE-derived putative genes for CHH contexts (Wilcoxon rank sum test, *P* < 0.05). This phenomenon is conceivable since promoters of MULE-derived putative genes tend to locate in both TIR and internal sequences of MULEs, which might lead to the mixed pattern of the two types of regions. Additionally, the methylome data were processed with a modified version of genomemapper (http://1001genomes.org/software/genomemapper.html) which only used reads with unique genomic targets. Therefore, this method excluded the possibility that the lower methylation levels detected in the younger genic-MULEs resulted from mis-counting reads from their homologous parental sequences, which are generally lowly methylated.

**Fig. 6:**
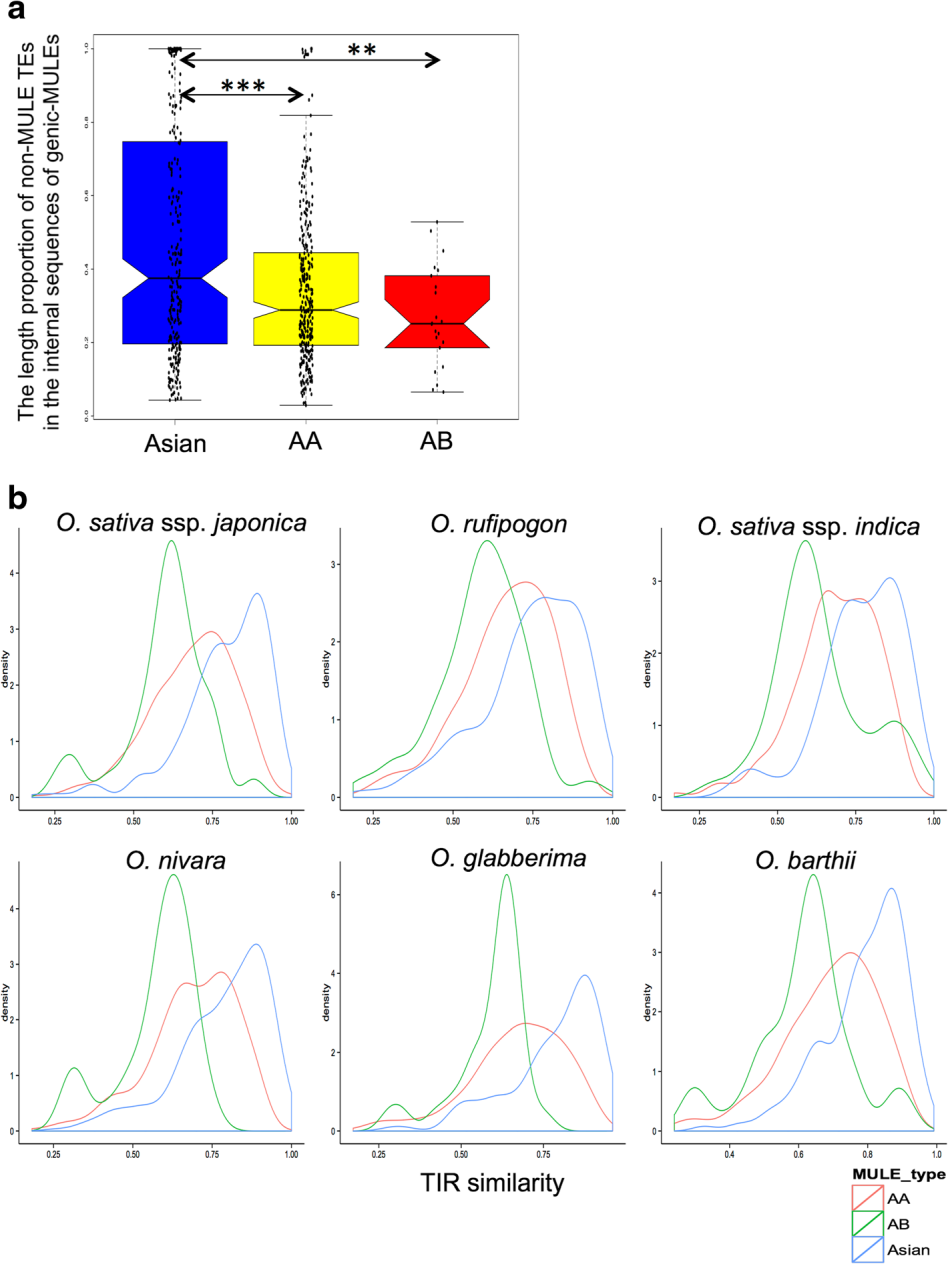
Validation of dynamic methylation patterns of genic-MULEs. **a** Distributions of TE content inside the genic-MULE internal sequences with three evolutionary ages. This analysis shows that older genic-MULEs have significantly lower or similar TE content compared with younger elements (Wilcoxon rank sum test, *P* = 3.006e-5 for Asian & AA, and *P* = 0.004681 for Asian & AB). **b** Density distribution of TIR similarity of genic-MULEs with three evolutionary ages in each domesticated *Oryza* species (*O. sativa* ssp*. japonica*, *O. sativa* ssp*. indica*, and *O. glaberrima*) and their wild progenitors (*O. nivara*, *O. rufipogon*, and *O. barthii*, respectively). TIR similarity decreases over time (Wilcoxon rank sum test, *P* < 0.003). **: *P* < 0.01, ***: *P* < 0.001

It could be argued that the higher methylation levels observed in older MULE internal sequences could be achieved by the accumulation of highly methylated TEs inserted into older MULEs. To test this possibility, we measured TE content within genic-MULEs (defined as the proportions of non-MULE TEs in the internal sequences of genic-MULEs) for the above three evolutionary ages of genic-MULES. This analysis revealed that the TE content in the older genic-MULEs was actually significantly lower than, or similar to, that found in the younger elements (the TE content of Asian genic-MULEs was greater than that of AA genic-MULEs; that of Asian genic-MULEs was greater than that of AB genic-MULEs; and that of AA genic-MULEs is not significantly different from that of AB genic-MULEs; Wilcoxon rank sum test, *P* = 3.006e-5 and *P* = 0.004681; Fig. 6a). Thus, this result rejected the possibility that the higher methylation levels observed in older MULE internal sequences were caused by the accumulation of higher TE contents in older MULEs.

To investigate the observed reduction of TIR methylation levels in a CHH context, we hypothesized that a decrease in TIR similarity over evolutionary time may be the cause. As demonstrated earlier, highly diverged TIRs could have two consequences. First, the transposase could not recognize the TIRs flanking the TEs and thus could not excise TEs out of their donor positions. Consequently, TEs might lose mobility. Second, TEs with divergent TIRs might not be able to fold back to form hairpin structures; thus double-stranded RNAs might not be processed into small interference RNAs (siRNAs) to induce de novo DNA methylation in a CHH context. Consequently, TIRs could not be recognized by asymmetrical methylation machinery [35]. To test this hypothesis, we examined the similarity of the two copies of paired TIRs for each of the domesticated *Oryza* species *O. sativa* ssp*. japonica*, *O. sativa* ssp*. indica*, and *O. glaberrima* with their wild progenitors *O. nivara*, *O. rufipogon*, and *O. barthii*, respectively, in genic-MULEs with the aforementioned three evolutionary ages. Indeed, TIR similarity decreased over evolutionary time as TIR similarity of Asian/African genic-MULEs was greater than that of AA genic-MULEs which was greater than that of AB genic-MULEs (Wilcoxon rank sum test, *P* < 0.003; Fig. 6b). Furthermore, the gradient of TIR similarity over time also demonstrated that the sample of MULEs with different evolutionary ages was not biased toward MULEs with the same criteria of TIR similarity in our MULE identification methods; thus, we did include cases of older MULEs with more divergent TIRs.

However, we might have included more false positive MULEs in either older or younger MULEs from our identification approach, leading to the above trends. To test this possibility, we re-performed the above analyses using the overlapping *O. sativa* ssp*. japonica* genic-MULEs from a previous study [7] and from our study. The patterns of methylation, TE coverage, and TIR similarity all maintained similar trends, excluding the possibility of biased inclusion of false positives in older or younger MULEs (Additional file 1: Figures S3b, S4, and S5). To test whether our observed patterns are *O. sativa* ssp. *japonica* species-specific, we conducted the above analyses for *O. nivara* genic-MULEs. The patterns of methylation, TE coverage, and TIR similarity all had similar trends, suggesting this is a general behavior of genic-MULEs in *Oryza* genomes (Additional file 1: Figures S3c, S6, and S7). We further tested the reliability of our results with the BS-seq data generated by Li et al. [36] and found similar trends (data not shown), suggesting the robustness of our results. Overall, these results imply that the evolution of genic-MULEs and MULE-derived putative genes is associated with dynamic DNA methylation levels in MULE internal and TIR regions.

### Methylation of genic-MULEs directed through small RNA-mediated pathways

It has been shown that small RNAs trigger the methylation of *MuDR* in maize and that 24-nucleotide small RNAs are the most abundant small RNA species that can induce DNA methylation [37–40]. To track the possible mechanism regulating the methylation of MULE internal sequences and TIRs, we identified 24-nucleotide small RNA occupancy in the internal and TIR regions of the three groups of genic-MULEs, with the aforementioned evolutionary ages, in 12 tissues/conditions of *O. sativa* ssp. *japonica*, including tricellular pollen (TCP), bicellular pollen (BCP), uninucleate microspores (UNMs), callus, leaf, seedling, root, shoot, panicle, two RNA interference lines, and one wild-type plant. It has been demonstrated that a considerable subset of RNA-mediated DNA methylation might be directed by multiple mapping small RNA, which contains multiple mapping locations in the genome. However, a multiple mapping small RNA does not induce methylation for all homologous loci [41]. Here, we attempted to study the causal relationships between DNA methylation and small RNA, so our analyses mainly focused on unique-mapping small RNAs to avoid mis-association between small RNAs and their true activity location [41, 42].

**Fig. 7:**
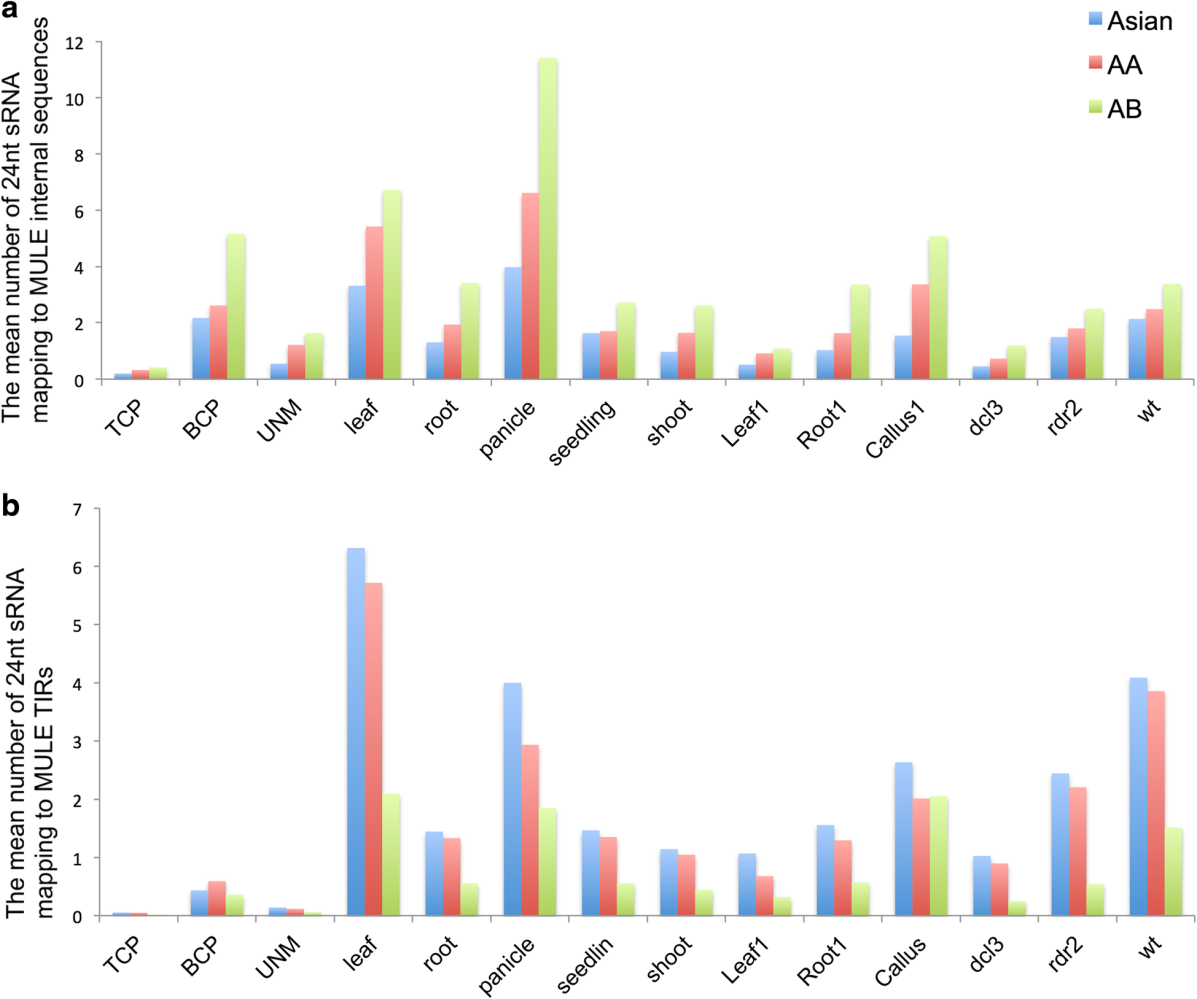
Mean number of 24-nucleotide small RNAs uniquely mapping to genic-MULE internal sequences and TIRs in the *O. sativa* ssp. *japonica* genome. **a** Mean number of 24-nucleotide small RNAs uniquely mapping to genic-MULE internal sequences in nine tissues and one wild-type (*wt*) and two RNA interference lines. The number of 24-nucleotide small RNAs increases over time (*t*-test, *P* < 0.002), which is consistent with the change in methylation levels in genic-MULE internal sequences over time. **b** Mean number of 24-nucleotide small RNAs uniquely mapping to genic-MULE TIRs. The number of 24-nucleotide small RNAs decreases over time (*t*-test, *P* < 0.004), which is consistent with the dynamics of methylation levels in genic-MULE TIR regions over time

Interestingly, the mean number of 24-nucleotide small RNAs uniquely mapping to MULE internal sequences was found to increase over evolutionary time in the 12 tissues/conditions tested (the mean number of 24-nucleotide small RNAs uniquely mapping to the internal sequences of Asian genic-MULEs was less than that of AA genic-MULEs which was less than that of AB genic-MULE, *t*-test, *P* < 0.002; Fig. 7a), whereas the mean number of 24-nucleotide small RNAs uniquely mapping to MULE TIR regions decreased over evolutionary time (the mean number of 24-nucleotide small RNAs uniquely mapping to the TIR regions of Asian genic-MULEs was greater than that of AA genic-MULEs which was greater than that of AB genic-MULEs, *t*-test, *P* < 0.004; Fig. 7b). Further, the lengths of older genic-MULE internal sequences is similar to or shorter than those of younger genic-MULEs (the lengths of AA genic-MULE and AB genic-MULE internal sequences are significantly shorter than the lengths of Asian genic-MULE internal sequences, Wilcoxon rank sum test, *P* < 0.05, and the lengths of AA genic-MULE internal sequences are similar to those of AB genic-MULE internal sequences). Therefore, the observation that the mean number of 24-nucleotide small RNAs uniquely mapping to the internal sequences of older genic-MULEs is higher than that of younger genic-MULEs is not due to the longer length of the internal sequences of older genic-MULEs. For TIR regions, since they usually have similar length, the length factors should not affect the decreasing mean number of 24-nucleotide small RNAs uniquely mapping to the TIRs over time. When considering unique and multiple mapping small RNAs together, the mean number of 24-nucleotide small RNAs mapping to MULE TIR regions decreased over time, similar to the aforementioned trend (*t*-test, *P* < 0.005). Thus, the change in 24-nucleotide small RNA abundance in the internal and TIR regions of genic-MULEs over time is consistent with the dynamic methylation levels in these regions over time presented above, suggesting that methylation of MULEs might be directed by the 24-nucleotide small RNA-mediated pathway.

### Association of biased expression of MULE-derived putative genes with developmental de-methylation in mature pollen

**Fig. 8:**
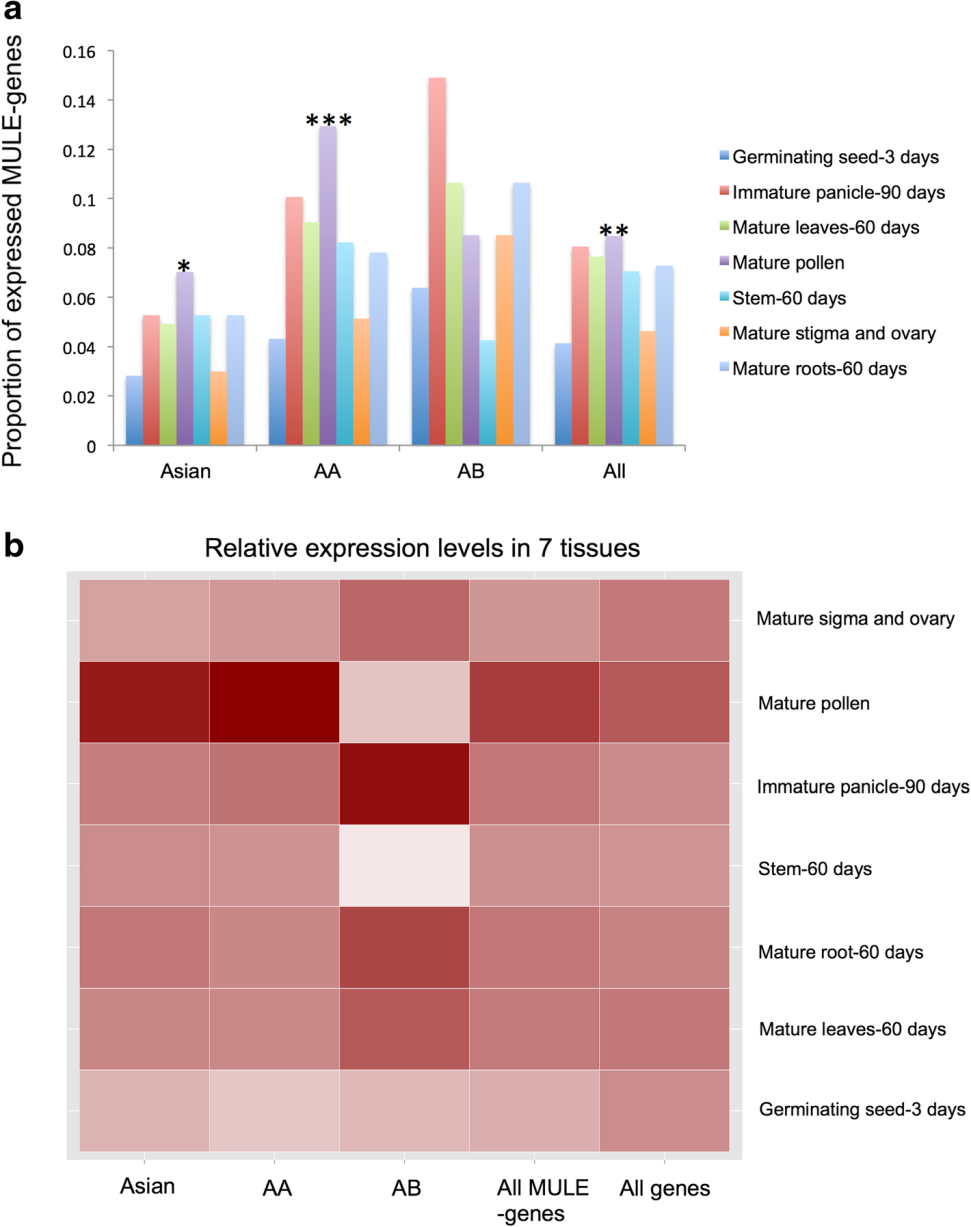
Expression profile of MULE-derived putative genes in *O. sativa* ssp. *japonica*. **a** Proportion of MULE-derived putative genes expressed across seven tissues with different evolutionary ages (Asian genic-MULEs, AA genic-MULEs, AB genic-MULEs, and all genic-MULEs). A higher proportion of the MULE-derived putative genes are expressed in mature pollen compared with all annotated *Oryza* genes (Fisher exact test, *P = 0.0183 for Asian MULE-derived putative genes, ***P = 0.0006463 for AA MULE-derived putative genes, **P = 0.001295 for all MULE-derived putative genes). **b** Heatmap of relative gene expression levels for MULE-derived putative genes versus all annotated *Oryza* genes. These data suggest that MULE-derived putative genes, especially those associated with younger genic-MULEs, are highly expressed in mature pollen compared with all annotated *Oryza* genes (note that the data size of expressed MULE-derived putative genes from AB genic-MULEs is very small, with only 17 genes detected, which might not, therefore, represent a statistically significant pattern)

Previous studies have shown that new genes in *Arabidopsis* tend to be transcribed in mature pollen [43, 44]. This intriguing observation led us to ask whether MULE-derived putative genes in *Oryza* also have biased tissue expression patterns. We focused on the expression patterns of MULE-derived putative genes in *O. sativa* ssp. *japonica*, which have comprehensive expression profiles from multiple tissue and developmental stages (i.e., 3-day seed, 60-day mature leaf, 60-day mature root, 60-day stem, 90-day immature panicle, mature pollen, mature stigma, and ovary). Expression profiles of MULE-derived putative genes from Asian genic-MULEs, AA genic-MULEs, AB genic-MULEs, and all genic-MULEs were compared with all *Oryza* annotated genes. Interestingly, the proportion of MULE-derived putative genes expressed in mature pollen was higher than that of all *Oryza* annotated genes (Fig. 8a; Fisher exact test, *P* = 0.0183 for Asian MULE-derived putative genes, *P* = 0.0006463 for AA MULE-derived putative genes, *P* = 0.001295 for all MULE-derived putative genes).

We then examined the relative expression levels among the seven tissues for genes that were expressed in at least one tissue and also found that MULE-derived putative genes, especially those from younger genic-MULEs, have higher expression levels in mature pollen compared with all *Oryza* annotated genes (Fig. 8b). Further, by integrating the expression profiles with the small RNA analysis described above, we observed that mature TCP tissue had the lowest 24-nucleotide small RNA occupancy in all three age groups of genic-MULE internal and TIR regions compared with all other tissues tested (Fig. 7). If 24-nucleotide small RNAs mediate the methylation of genic-MULEs, the lack of 24-nucleotide small RNAs in TCP might be associated with the de-methylation of TCP, which might lead to the mature pollen-biased expression pattern we observed for MULE-derived putative genes. This is also consistent with the fact that the vegetative nucleus in TCP undergoes a developmental de-methylation reprogramming stage [38, 45], which could provide a more favorable expression environment for newly formed/TE-derived genes.

## Discussion

### Rapid turnover of non-autonomous MULEs and MULE-derived gene origination

The genus *Oryza* contains 24 species that have been classified into 11 distinct genome types, represented by six diploids and four allotetroploids [46], that vary over a 3.6-fold genome size range, i.e., from 362 Mb (*O. brachyantha*) to 1283 Mb (*O. ridleyi*). Genomic analyses across the genus have demonstrated that the heterogeneous size and evolution of the *Oryza* genomes are largely affected by TE proliferation/elimination and polyploidy [47, 48]. Using a recently generated high-quality 11 genome dataset of closely related *Oryza* species, we systematically and comprehensively generated a unique genus-wide vertical database of MULEs and investigated their evolutionary and sequence capture history over a ~20 million year time frame.

The distribution and amount of MULE accumulation identified across the *Oryza* and *Leersia* genome dataset revealed several general patterns. First, the number of MULEs identified in the basal genomes of *L. perrieri* and *O. brachyantha* (Fig. 1) was ~600 each, whereas the remaining nine *Oryza* species contained three- to fivefold as many elements. This suggests that MULE proliferation was relatively passive in the basal species and active across the majority of the *Oryza* species tested or that fewer MULEs have survived in the basal species. Second, the ratio of genic- to nongenic-MULEs in *L. perrieri* and *O. brachyantha* is very low compared with those in other *Oryza* species (Fig. 1). This difference may be impacted by genome size. The numbers of genic-MULEs in the 11 genomes seem to positively correlate with genome size (Pearson correlation coefficient = 0.6689487, *P* = 0.0244) but those of nongenic-MULEs do not (Pearson correlation, *P* > 0.05). The basal species have smaller genome sizes compared with the other *Oryza* species, which might explain the lower proportion of genic-MULEs in their genomes. Third, the number of MULEs in the internal branches of the phylogenetic tree is much lower than that in the terminal species nodes (Fig. 2), revealing that MULE sequences were very rapidly removed if not selected for or might not be recognizable after a long evolutionary time. Fourth, incomplete lineage sorting has been proposed as a general phenomenon for the evolution of TEs [49]. However, the consistency among the origination time of MULEs inferred from the phylogenetic tree, the amplification time of MULEs inferred from sequence divergence of MULEs, and the speciation time of the *Oryza* species (Additional file 1: Figure S1) suggest that incomplete lineage sorting may not play a major role in the evolution of non-autonomous MULEs, although it could partially explain it.

New genes can be generated through various mechanisms [18, 19, 21, 22]. Previous studies indicate that Pack-MULEs can serve as “vehicles” for the formation of potentially functional genes in *O. sativa*. We identified thousands of potentially functional putative genes that arose rapidly and evolved from non-autonomous MULE sequences in *Oryza*. By defining the species-specific origination of MULE-derived putative genes, we determined that new genes originating from non-autonomous MULEs could be one of the main sources of new gene origination in *Oryza*. More interestingly, MULE-derived putative genes tended to arise from multiple parental sequences, which could potentially form novel chimeric gene structures. This is consistent with our previous discovery that the formation of chimeric ORFs is the general mode of new gene origination in *Oryza* species [15, 50]. We also observed that only a small proportion of MULE-derived putative genes show functional constraints based on Ka/Ks ratios, suggesting the majority of MULE-derived putative genes evolved neutrally and could proceed rapidly to extinction due to genetic drift after a certain evolutionary time period. However, approximately 100 MULE-derived putative genes in most *Oryza* species show evidence of natural selection, suggesting a notable amount of MULE-derived putative genes could play functional roles in the evolution of *Oryza* genomes and species.

Previous studies have shown that MULEs are able to redistribute GC-rich sequences to affect the GC gradient of genes within some monocot genomes [7]. By extending our analysis to ten closely related *Oryza* and one *Leerisa* species, we consistently demonstrated that MULE-derived putative genes are GC-rich, especially in the parts derived from MULE internal sequences, which are derived from GC-rich parental sequences selectively captured by MULEs. Remarkably, we also found that MULEs tend to acquire sequences from genomic regions with low methylation levels and high recombination rates, which might provide a more open chromatin structure that could promote the invasion/conversion of MULEs in the process of acquiring parental sequences.

### DNA methylation might facilitate the survival of MULE-derived genes

We showed that the methylation level in three cytosine contexts of MULE internal sequences increases and that the CHH context of MULE TIRs decreases over evolutionary time. This result implies that genic-MULEs which acquired potentially functional coding sequences and were maintained in the multiple *Oryza* species over millions of years might acquire epigenetic marks in their internal and TIR sequences that are needed to maintain their stability in the genome. That is, methylation marks present in MULE internal sequences might reduce chromatin structure accessibility and ensure transcription, whereas reduced methylation marks in TIRs might indicate low sequence similarity and decreased mobility of these TIRs [35, 51–53]. Overall, the dynamics of DNA methylation levels in the internal and TIR regions of genic-MULEs might facilitate the evolution of MULE-derived genes.

A survey of small RNA occupancy in genic-MULEs suggested that 24-nucleotide small RNAs might mediate the methylation of MULE internal sequences and TIRs through the RNA-mediated DNA methylation pathway. Moreover, we showed that MULE-derived putative genes tend to be transcribed in mature pollen. This interesting phenomenon coincides with testis-biased expression patterns of new genes in *Drosophila* and mammals and is also consistent with the “out of pollen” expression pattern of *Arabidopsis* new genes, all of which are related to reproductive tissues. Both testis and pollen potentially provide an open chromatin structure that results from developmental chromatin remodeling in these tissues and is permissive for gene expression [43, 45, 54]. Further, we found a consistent pattern whereby genic-MULEs have the lowest occupancy of 24-nucleotide small RNAs in TCP, where vegetative pollen cells experience overall de-methylation and the loss of CG methylation [43, 45], suggesting the involvement of 24-nucleotide small RNAs in the regulation of DNA methylation. These results further support that pollen-biased expression of MULE-derived genes may be related to the developmental epigenetic reprogramming of reproductive tissues, which could help to promote the expression of newly formed/TE-derived genes.

## Conclusions

Our results suggest that DNA methylation may play an important role in the origination and survival of MULE-derived genes through modulation of their stability and expression, which might be a general mechanism for all the TE-derived genes, thereby contributing to the evolution of gene novelty. Further experimental studies should be conducted in this area to explore and demonstrate the causal logistics between DNA methylation and the evolution of TE-derived new genes.

## Methods

### Plant genomes, transcriptomes, methylomes, and annotation data

The complete set of genome sequences and “gff” MAKER [55] annotation files for ten *Oryza* species and *L. perrieri* were downloaded from the iPlant Collaborative (iPlant) data store (http://data.iplantcollaborative.org/) hosted by the Arizona Genomics Institute (AGI) at the University of Arizona. Plant TE and repeated sequence (PReDa) libraries were generated by AGI [56]. Baseline RNA-seq data were generated by AGI from three tissues, including leaf, root, and panicle, from nine *Oryza* and one *Leersia* species, including *O. sativa* ssp. *japonica*, *O. nivara*, *O. rufipogon*, *O. glaberrima*, *O. barthii*, *O. glumipatula*, *O. meridionalis*, *O. punctata*, *O. brachyantha*, and *L. perrieri*. The raw digital gene expression (DGE) reads derived from seven *O. sativa* ssp. *japonica* tissues were downloaded from the NCBI Short Read Archive (http://www.ncbi.nlm.nih.gov/sra/) with accession numbers SRR074144 (germinating seed at 3 days), SRR074145 (immature panicle at 90 days), SRR074146 (mature leaves at 60 days), SRR074147 (mature pollen), SRR074151 (stem at 60 days), SRR074170 (mature stigma and ovary), and SRR074171 (mature roots at 60 days) [57]. Processed small RNA data from *O. sativa* ssp*. japonica* in 12 tissues or conditions were downloaded from https://mpss.danforthcenter.org/dbs/index.php?SITE=rice_sRNA, which lists the codes (with original references) of the downloaded tissues or conditions, including leaf, root, BCP, callus, TCP, UNM [58], SC1I (seedling), RCn2D (root), ShCn2D (shoot), PC1C (panicle) [59], WT2003s (leaf) [60], *dcl3*_*sdl*, *rdr2*_*sdl*, and *wt*_*sdl* [61]. Processed BS-seq data were downloaded from the iPlant Collaborative (iPlant) data store.

### Identification of MULEs, genic-MULEs, nongenic-MULEs, and MULE-derived putative genes

The annotated *Oryza sativa* ssp. *japonica* MULE TIR sequences were obtained from Ferguson et al. [7]. To identify de novo MULE TIR families, RepeatScout (version 1.0.5) [62] was used to scan all ten *Oryza* and one *Leerisa* genomes. Repeat families identified by RepeatScout with at least 20 copies in the genome were collected and grouped with consensus sequences of the known *O. sativa* MULE TIR sequences. To remove the sequences of other known non-MULE repeats from the merged repeat sequence dataset (described above), RepeatMasker (A.F.A. Smit, R. Hubley, and P. Green, http://repeatmasker.org) was used to mask the merged sequences with plant repeat sequences (i.e., the classified PReDa sequences) as the library [56]. Repeat sequences with ≥30 % of the length masked by known non-MULE elements from PReDa [56] were discarded. To determine whether each de novo consensus sequence represented a MULE TIR, RepeatMasker was run on all 11 genome sequences with the above remaining TIR sequences as the library.

Putative MULE TIRs were identified/called if two repeats satisfied the following criteria: (1) the two repeats belonged to the same family; (2) the ends were in opposite orientations; (3) the distance between the two repeats was less than 20 kb; (4) a 7–11 bp tandem TSD lay immediately adjacent to both repeats (we allowed a maximum of two mismatches/indels in the 9–11-bp TSDs, one mismatch/indel in 8-bp TSDs, and a perfect match in 7-bp TSDs) [9, 13]. If a de novo consensus sequence had at least five such elements identified, this sequence was classified as a MULE TIR. Then, for each of the 11 genomes, the identified de novo MULE TIRs and *O. sativa* MULE TIRs were grouped together to mask all genomes with RepeatMasker to identify MULE elements. Complete MULEs contained two TIRs embracing an internal sequence and we required a tandem TSD flanking each TIR of a MULE. TSDs were allowed to have a maximum of a 10-bp swing from the putative ends of each TIR [7]. Next, we used TBLASTN on annotated transposase proteins and *Mutator* transposases collected from NCBI against MULE sequences. MULEs that contained transposase proteins/*Mutator* transposases (TBLASTN E value <1e-9) were removed. All remaining MULEs were defined as “non-autonomous MULEs”.

To identify genic-MULEs, we examined whether non-autonomous MULE sequences overlapped with annotated ORFs. We compared the genomic coordinates of non-autonomous MULEs with the coordinates of transcripts annotated with MAKER (Stein et al., in preparation). For MULEs that did not overlap with the MAKER annotated genes, GlimmerHMM was used to annotate potential gene structures within them. If a MULE overlapped by at least 30 % the length of a MAKER/GlimmerHMM annotated transcript which had at least 150 bp of coding sequence, including intact start and stop codons, and did not carry any transposases, the MULE was annotated as a “genic-MULE”. Overlapping putative genes with at least 150 bp of coding sequence and intact start and stop codons (annotated by MAKER/GlimmerHMM) were defined as MULE-derived putative genes. Finally, non-autonomous MULEs that did not carry any transposases or potential gene fragments were annotated as “nongenic-MULEs”.

### Identification of the presence and absence of MULEs in 11 genomes

Using one genome, both identity and local syntenic evidence were used to determine the presence of non-autonomous MULEs in each of the ten other genomes (excluding its own genome). Sequences in upstream and downstream 2-kb windows flanking MULEs together with MULE sequences (i.e., 1 bp–1 kb upstream, 1–2 kb upstream, 1 bp–1 kb downstream, 1–2 kb downstream, and MULE sequences) were collected from each of the 11 genomes and used to probe each of the ten other genomes using BLAT [63]. This process was done iteratively for each single species. To satisfy our identity criterion, a MULE sequence was required to have the best BLAT hit in the other species with at least 30 % coverage of its entire length.

For synteny evidence, a MULE sequence was required to satisfy at least one of the following two criteria: (1) the best BLAT hit of the MULE sequence in other species was located on the same chromosome as the one in its own species and the best BLAT hit of at least one flanking sequence was located within 4 kb upstream or downstream of the best BLAT hit of the MULE sequence in the other species; or (2) the best BLAT hits of at least two flanking sequences were located within 4 kb upstream or downstream of the best BLAT hit of the MULE sequence in the other species. If the best BLAT hit of a MULE passed both of the above identity and syntenic criteria, we inferred that the MULE under investigation was present in other species.

Based on the presence or absence information of each MULE in the 11 species, the evolutionary parsimony principle, and the phylogenetic tree of the 11 species [46], the origination time point of a MULE to an external species or internal branches was assigned (Fig. 2). For example, (1) if a MULE was identified in only one species but not the other ten species, it was annotated as a species-specific MULE. (2) If a MULE was identified in both *O. sativa* ssp. *japonica* and *O. rufipogon*, but not another species, it was inferred that it originated before the divergence of *O. sativa* ssp. *japonica* and *O. rufipogon* but after the split of this branch from the rest of the *Oryza* species. (3) If a MULE was found in all the Asian *Oryza* species but not the other species, it was inferred that it originated before the divergence of Asian species but after the split of Asian species from the rest of the *Oryza* species. (4) If a MULE was only present in all AA genome *Oryza* species but not the other species, it was inferred that it originated before the divergence of AA genome *Oryza* species but after the split of AA genome *Oryza* from BB genome *Oryza* (as an AA-MULE). We collected the number of non-autonomous MULEs at each evolutionary time point and listed them in the phylogenetic tree of the 11 genomes (Fig. 2).

To validate the above origination time (age) assignment of non-autonomous MULEs, we estimated the amplification time of each MULE. We computed the amplification time of each MULE based on the sequence divergence of each non-autonomous MULE and its most similar paralogous non-autonomous MULE that belonged to the same MULE TIR family [28]. We conducted all-by-all BLAT searches of all non-autonomous MULEs for each species. Each MULE would then be aligned with its second best hit with the same MULE TIR, followed by a calculation of the corresponding sequence divergence using the baseml module of PAML (version 4.7) [32]. Based on the formula T = k/2r, where k = sequence divergence and r = substitution rate, and calibrating with r = 1.3 × 10^-8^ per site per year for rice [28], we computed the amplification time of each MULE. MULEs were categorized based on their origination time points, inferred from the presence and absence of MULEs in the *Oryza* phylogenetic species tree, and we drew the density distribution of the amplification time of MULEs in each origination time point category for the 11 species.

### Identification of species-specific MULE-derived putative genes

Gene and protein sequences of MULE-derived putative genes associated with species-specific genic-MULEs were extracted for each genome and we used BLAT or BLASTP to identify homologous sequences in the other ten genomes. If the coordinates of the best BLASTP protein hit (with BLASTP E-value <1e-10) of a MULE-derived putative gene in another species overlapped with the coordinates of its best BLAT genomic sequence hit in the same species, we assumed the presence of the MULE-derived putative gene in the other species. If a gene had a best BLAT genomic sequence hit in another genome but the coordinates did not overlap with the best BLASTP protein hit in the same genome, or the best BLASTP protein hit did not exist (e.g., did not satisfy BLASTP E-value <1e-10), the genomic sequence of the best BLAT genomic sequence hit in the other genome was extracted and annotated with Glimmer. Then the peptide sequence of the MULE-derived putative gene was used as a probe against the Glimmer-annotated peptide sequence using BLASTP. If the BLASTP E value was <1e-10, we assumed that the MULE-derived putative gene was present in the other species. For the remaining cases, we assumed that the MULE-derived putative gene was not present in the other species. If a MULE-derived putative gene was absent in all ten species, we annotated it as a species-specific MULE-derived putative gene.

### Identification of the parental sequences of MULE-derived putative genes

For each species, non-MULE TE sequences from the plant repeat sequence library (PReDa [56]) were used to mask MULE-derived putative gene sequences with RepeatMasker and then BLASTN was used to map the masked MULE-derived putative gene sequences against the corresponding whole-genome sequence. We also used TE and MULE TIR sequences to mask the corresponding whole genome sequence with RepeatMasker to generate TE and MULE TIR coordinates of the genome. The coordinates of the BLASTN hits of MULE-derived putative gene sequences were then compared with those of MULE TIRs and TEs. BLASTN hits that were not flanked with MULE TIRs and not associated with TEs and had the highest identity score (with BLASTN E value <1e^-10^) were annotated as parental sequences of MULE-derived genes [13]. The genomic coordinates of these parental sequences were further compared with MAKER annotated genes. If the coordinates of the parental sequences overlapped with those of the MAKER genes, the MAKER genes were classified as the corresponding parental genes [13].

### Ka/Ks computation

Ka/Ks ratios between the CDS of MULE-derived putative genes and their closest paralogous non-autonomous MULE sequences were computed using a modified gKaKs pipeline [28, 31]. We used the CDS of MULE-derived putative genes to query all the non-autonomous MULEs using BLAT. Each MULE-derived putative gene CDS was then paired with its most similar MULE sequences with the same MULE TIR. Lastly, we computed Ka/Ks ratios of paired sequences with the modified gKaKs pipeline using the Codeml option from PAML [31, 32]. This pipeline can handle the Ka, Ks, and Ka/Ks calculations between one CDS sequence and an un-annotated genomic sequence by automatically removing frame-shift and premature stop codons in the sequence alignment. We estimated Ka/Ks with two Codeml models: (1) Ka/Ks varying freely and (2) Ka/Ks fixed at 1 (neutrality). Tests for significant difference (*P*) between two models were calculated using the likelihood ratio test, where the test statistic is 2Δl = 2 × (l_1_ − l _2_) with l_1_ and l_2_ as the log of the maximum likelihood (ML) estimated from the two models compared. It is assumed that 2Δl is approximately distributed as *X*^2^ with difference of model parameters as degrees of freedom (d.f.). We then computed the corresponding q value, namely the false discovery rate, for each P value of the likelihood ratio test using the qvalue package of R. A q value of ≤ 0.05 was used as the significance cutoff [64, 65].

### Estimation of the expression of MULE-derived putative genes

RNA-seq reads from leaf, root, and panicle of nine *Oryza* and one *Leersia* species were mapped to their corresponding genomic regions with TopHat and FPKM was computed with Cufflinks. FPKM values were then mapped to the genes of interest. If the overlapping length between a FPKM region and the gene of interest was ≥50 % of the gene length, the FPKM value was assigned to the gene as the “expression intensity”.

### Generation of non-TE genes

We mapped the plant repeat sequence library (PReDa [56]) and MULE TIR sequences to each of the 11 genomes with RepeatMasker. According to the coordinates of TEs and MULE TIRs and the coordinates of MAKER-annotated genes, we removed the genes which overlapped or were flanked (in 500-bp/1000-bp flanking region) by TE or MULE TIR sequences. We considered the remaining genes as non-TE genes.

### Estimation of recombination rate

To measure recombination rates, a genetic versus physical distance map (Marey’s map) was constructed using 1673 markers of the rice genetic map available at http://cgpdb.ucdavis.edu/XLinkage/genetic_map_rice/. All available 5′ and 3′ probe sequences of all markers from the above website were mapped to the *O. sativa* ssp*. japonica* cDNA sequences using BLASTN (since the markers came from cDNA) [66]. Best hit cDNAs with a BLASTN E value ≤1e-^10^ were selected and the midpoint of each cDNA was used as the physical distance of the mark. Marks that had multiple positions in the genome and/or anomalous positions after visual inspection of the Marey’s maps were removed. Based on both genetic and physical distances of these marks, Marey’s maps were built using the MareyMap program [67]. To compute the interpolation and generate a recombination rate map of the *O. sativa* ssp. *japonica* RefSeq, we used the LOESS function with a window span size of 20 %, a fitted curve degree of 2, and the cubic splines method with the cross-validation option (Additional file 1: Figure S8) [55]. Based on the recombination rate map, we estimated local recombination rates of the parental sequences of MULE-derived putative genes and non-TE genes using the MareyMap program [67]. Both the recombination rate and Marey’s maps are shown on Additional file 1: Figure S8.

### Methylome data processing

The methylome BS-seq raw data of *O. sativa.* ssp. *japonica* and *O. nivara* genomes were processed according to Becker et al. [68]. For methylation analyses, we only considered cytosine sites covered by at least three BS-seq reads. The methylation level of a region was estimated as the percentage of methylated cytosines over the total number of mapped cytosines in that region for the three cytosine contexts (CG, CHG, and CHH), respectively. Only regions where at least 50 % of the cytosines were mapped were considered. Thus, we estimated the methylation levels of the parental sequences of MULE-derived putative genes and the randomly selected sequences (with the same size as the mean size of the parental sequences) from non-TE genes in the three cytosine contexts and compared the methylation levels of the two groups of sequences with the Wilcoxon rank sum test. Further, we categorized and computed the methylation levels in internal, TIR, and 500-bp flanking regions of genic-MULEs with three evolutionary ages (Asian genic-MULEs, AA genic-MULEs, and AB genic-MULEs) and compared the methylation levels of the three groups of genic-MULEs with the Wilcoxon rank sum test. Methylation levels were also calculated and compared in the gene body and promoter regions of MULE-derived putative genes over three evolutionary ages. We also analyzed the methylation patterns with increased BS-seq read coverage, considering cytosine sites covered by at least five BS-seq reads and at least seven BS-seq reads, respectively. We found similar patterns as the ones considering cytosine sites covered by at least three BS-seq reads. Therefore, we only present the results based on the analysis using at least three BS-seq reads as the threshold of read coverage.

### Estimation of TE content of genic-MULE internal sequences

To estimate the TE content of genic-MULE internal sequences, we first removed MULE TIR sequences from PReDa [56]. The resultant repeat library was then mapped to MULE internal sequences using RepeatMasker. The length of masked MULE internal regions divided by the total length of MULE internal sequences was calculated as the TE content of MULEs. The TE contents of genic-MULEs with three evolutionary ages were computed and compared using the Wilcoxon rank sum test for the *O. sativa.* ssp. *japonica* and *O. nivara* genomes, respectively.

### Calculation of TIR identity

Paired TIR regions of MULEs were extracted and aligned with MAFFT [69]. TIR similarity was computed as the total number of identical bases divided by the length of the left TIR.

### Small RNA data processing

Small RNA sequences from 12 tissues or conditions of *O. sativa* ssp. *japonica* were mapped to the *O. sativa* ssp*. japonica* genome using BWA (bwa-12-17-2013-git) with perfect matches. The output from BWA (in SAM format) was parsed and the number of locations where each small RNA mapped to the genome was counted. We extracted small RNAs that mapped to only one location on the genome and considered these as unique targets in the genome for our analyses [41, 42]. Only 24-nucleotide small RNAs were considered since they are known to be able to induce DNA methylation [37, 38]. If the coordinates of 24-nucleotide small RNA sites overlapped with the TIR/internal region coordinates of genic-MULEs, we assume that these small RNAs mapped to the TIR/internal regions of genic-MULEs. Based on the number of each 24-nucleotide small RNA, we estimated how many 24-nucleotide small RNAs were mapped to the TIR/internal region of the genic-MULEs. Thus, in 12 tissues/conditions, we estimated the mean number of 24-nucleotide small RNAs mapped to the TIR/internal regions of the genic-MULEs with three evolutionary ages and compared the three groups of values with a *t*-test.

### Processing DGE data of *O. sativa* ssp. *japonica*

Raw DGE reads from seven *O. sativa* ssp. *japonica* tissues were mapped to the *O. sativa* ssp*. japonica* genome using the TopHat v2.0.10 package. DGE abundance was then measured in exonic regions of MULE-derived putative genes (i.e., FPKM values) using Cufflinks (v2.1.1).

### Open access

This article is distributed under the terms of the Creative Commons Attribution 4.0 International License (http://creativecommons.org/licenses/by/4.0/), which permits unrestricted use, distribution, and reproduction in any medium, provided you give appropriate credit to the original author(s) and the source, provide a link to the Creative Commons license, and indicate if changes were made. The Creative Commons Public Domain Dedication waiver (http://creativecommons.org/publicdomain/zero/1.0/) applies to the data made available in this article, unless otherwise stated.

### Ethics approval

No ethical approval was required.

### Availability of supporting data and materials

All the intermediate steps were carried out with custom PERL and R scripts. The source codes used are available at GitHub (https://github.com/FanLabWayneStateU/MULE-methylation). The detailed workflow of the analysis procedure can be found in Additional file 3: Supplementary file S1.

All genome assembly, transcriptome, and methylome data are publically available from the National Center for Biotechnology Information (NCBI) and/or iPlant Collaborative (http://www.iplantcollaborative.org/). Accession numbers and URLs can be found in Additional file 4: Supplementary file S2.

## Additional files

Additional file 1: Figures S1. a) shows the phylogenetic tree of 10 *Oryza* and 1 *Leersia* species. Based on the presence and absence of MULEs on the phylogenetic tree, we inferred the origination time points of the MULEs measured with divergence time of the 11 species. We listed the origination time index on the phylogenetic tree as 1-9, e.g. “1” represents MULEs originated after each species diverged, “4” represents MULEs originated before Asian rice diverged but after Asian rice split from the rest of *Oryza* species, 9 represents MULEs originated before the AA and BB genome rice diverged. Then for each of the 9 *Oryza* 2 species, we grouped their MULEs based on its origination time index. b) We categorized MULEs based on their origination time points (shown in ‘a’) inferred from presence and absence of MULEs in the phylogenetic tree of *Oryza* species, and drew the density distribution of the amplification time of MULEs, which were estimated based on the sequence divergence of MULEs and their most similar paralogs, in each origination time point category for 9 species, respectively. **Figure S2.** Methylation level distribution of parental sequences of MULE-derived genes and randomly selected non-TE genic-regions in across the *O. nivara* genome. **Figure S3.** Methylation level of MULE-derived putative genes a) in *O. sativa* ssp. *japonica* genome; b) the genes derived from MULEs overlapped between Ferguson et al. and us.; c) in *O. nivara* genome. **Figure S4.** Analysis of methylation levels of genic-MULEs with the overlapped data set of Ferguson et al. and us. a) Methylation level of MULE internal sequences; b) Methylation level of entire MULEs and flanking regions. **Figure S5.** Analysis of TE-coverage and TIR similarity of genic-MULEs with the overlapped data set of Ferguson et al and us. **Figure S6.** Analysis of methylation levels of genic-MULEs in *O. nivara* genome. a) Methylation level of MULE internal sequences; b) Methylation level of entire MULEs and flanking regions. **Figure S7.** Analysis of TE-coverage of genic-MULEs in *O. nivara* genome. **Figure S8.** The Marey’s and recombination rate map of *O. sativa* ssp. *japonica* genome. The Blue line is based on LOESS function and the red line is based on cubic splines. (DOCX 3.19 mb) Additional file 2: Table S1. Estimation of the origination rate of MULE-derived candidate genes. (DOCX 14 kb) Additional file 3: Supplementary file S1. Detailed workflow for Wang et al. DNA methylation changes facilitated evolution of genes derived from *Mutator*-like transposable elements. (DOCX 156 kb) Additional file 4: Supplementary file S2. Accession numbers and URLs for genome assembly, transcriptome and methylome data that used in this project. (DOCX 101 kb)

## Abbreviations

AGI: Arizona Genomics Institute
BCP: bicellular pollen
CDS: coding sequence
DGE: digital gene expression
FPKM: fragments per kilobase of transcript per million reads
MULE: *Mutator*-like element
ORF: open reading frame
PReDa: plant TE and repeated sequences
TCP: tricellular pollen
TE: transposable element
TIR: terminated inverted repeat
TSD: target site duplication
UNM: uninucleate microspore
