# Supplementary material for: DNA methylation changes facilitated evolution of genes derived from Mutator-like transposable elements
Source: Genome Biol. 2016 May 6;17:92. doi: 10.1186/s13059-016-0954-8 (PMC4858842; doi:10.1186/s13059-016-0954-8)
Supplement: Additional file 1: Figures S1. — a) shows the phylogenetic tree of 10 Oryza and 1 Leersia species. Based on the presence and absence of MULEs on the phylogenetic tree, we inferred the origination time points of the MULEs measured with divergence time of the 11 species. We listed the origination time index on the phylogenetic tree as 1-9, e.g. “1” represents MULEs originated after each species diverged, “4” represents MULEs originated before Asian rice diverged but after Asian rice split from the rest of Oryza species, 9 represents MULEs originated before the AA and BB genome rice diverged. Then for each of the 9 Oryza 2 species, we grouped their MULEs based on its origination time index. b) We categorized MULEs based on their origination time points (shown in ‘a’) inferred from presence and absence of MULEs in the phylogenetic tree of Oryza species, and drew the density distribution of the amplification time of MULEs, which were estimated based on the sequence divergence of MULEs and their most similar paralogs, in each origination time point category for 9 species, respectively. Figure S2. Methylation level distribution of parental sequences of MULE-derived genes and randomly selected non-TE genic-regions in across the O. nivara genome. Figure S3. Methylation level of MULE-derived putative genes a) in O. sativa ssp. japonica genome; b) the genes derived from MULEs overlapped between Ferguson et al. and us.; c) in O. nivara genome. Figure S4. Analysis of methylation levels of genic-MULEs with the overlapped data set of Ferguson et al. and us. a) Methylation level of MULE internal sequences; b) Methylation level of entire MULEs and flanking regions. Figure S5. Analysis of TE-coverage and TIR similarity of genic-MULEs with the overlapped data set of Ferguson et al and us. Figure S6. Analysis of methylation levels of genic-MULEs in O. nivara genome. a) Methylation level of MULE internal sequences; b) Methylation level of entire MULEs and flanking regions. Figure S7. Analysis of TE-coverage of genic-MU [file 13059_2016_954_MOESM1_ESM.docx]

**Additional file 1:**

**Figure S1.** a) shows the phylogenetic tree of 10 *Oryza* and 1 *Leersia* species. Based on the presence and absence of MULEs on the phylogeny tree, we inferred the origination time points of the MULEs measured with divergence time of the 11 species. We listed the origination time index on the phylogeny tree as 1-9, e.g. “1” represents MULEs originated after each species diverged, “4” represents MULEs originated before Asian rice diverged but after Asian rice split from the rest of *Oryza* species, 9 represents MULEs originated before the AA and BB genome rice diverged. Then for each of the 9 *Oryza* species, we grouped their MULEs based on its origination time index.

b) We categorized MULEs based on their origination time points (shown in a) inferred from presence and absence of MULEs in the phylogeny tree of *Oryza* species, and drew the density distribution of the amplification time of MULEs, which were estimated based on the sequence divergence of MULEs and their most similar paralogs, in each origination time point category for 9 species, respectively

**Figure S2. Methylation level distribution of parental sequences of MULE-derived genes and randomly selected non-TE genic-regions in across the *O. nivara* genome**

**Figure S3. Methylation level of MULE-derived genes**

**a) in *O. sativa* ssp. *japonica* genome; b) the genes derived from MULEs overlapped between Ferguson et al. and us. ; c) in *O. nivara* genome.**

**Figure S4. Analysis of methylation levels of genic-MULEs with the overlapped data set of Ferguson et al. and us.** a) Methylation level of MULE internal sequences; b) Methylation level of entire MULEs and flanking regions.

**Figure S5. Analysis of TE-coverage and TIR similarity of genic-MULEs with the overlapped data set of Ferguson et al and us.**

**Figure S6. Analysis of methylation levels of genic-MULEs in *O. nivara* genome.** a) Methylation level of MULE internal sequences; b) Methylation level of entire MULEs and flanking regions.

**Figure S7. Analysis of TE-coverage of genic-MULEs in *O. nivara* genome.**

**Figure S8. The Marey’s and recombination rate map of *O. sativa* ssp. *japonica* genome. The Blue line is based on LOESS function and the red line is based on cubic splines method.**

**Chr1**

**Chr2**

**Chr3**

**Chr4**

**Chr5**

**Chr6**

**Chr7**

**Chr8**

**Chr9**

**Chr10**

**Chr11**

**Chr12**
