# Supplementary material for: DNA methylation changes facilitated evolution of genes derived from Mutator-like transposable elements
Source: Genome Biol. 2016 May 6;17:92. doi: 10.1186/s13059-016-0954-8 (PMC4858842; doi:10.1186/s13059-016-0954-8)
Supplement: Additional file 2: Table S1. — Estimation of the origination rate of MULE-derived candidate genes. (DOCX 14 kb) [file 13059_2016_954_MOESM2_ESM.docx]

| Species | Number of species-specific MULE-derived candidate genes | Species divergence time (MYA) | Origination rate of MULE-derived candidate genes |
| --- | --- | --- | --- |
| *O. sativa* ssp. *japonica* | 49 | 0.27 | 181.4814815 |
| *O. sativa* ssp. *indica* | 48 | 0.39 | 123.0769231 |
| *O. rufipogon* | 39 | 0.27 | 144.4444444 |
| *O. nivara* | 28 | 0.39 | 71.79487179 |
| *O. glaberrima* | 23 | 0.19 | 121.0526316 |
| *O. barthii* | 22 | 0.19 | 115.7894737 |
| *O. glumipatula* | 106 | 0.97 | 109.2783505 |
| *O. meridionalis* | 537 | 2.41 | 222.8215768 |
| *O. punctata* | 1293 | 6.76 | 191.2721893 |
| *O. brachyantha* | 238 | 15 | 15.86666667 |
| *Leersia perrieri* | 368 | 26 | 14.15384615 |

**Table S1. Estimation of the origination rate of MULE-derived candidate genes**
