# Supplementary material for: DNA methylation changes facilitated evolution of genes derived from Mutator-like transposable elements
Source: Genome Biol. 2016 May 6;17:92. doi: 10.1186/s13059-016-0954-8 (PMC4858842; doi:10.1186/s13059-016-0954-8)
Supplement: Additional file 3: — Supplementary file S1. Detailed workflow for Wang et al. DNA methylation changes facilitated evolution of genes derived from Mutator-like transposable elements. (DOCX 156 kb) [file 13059_2016_954_MOESM3_ESM.docx]

**Supplementary File S1**. **Detailed workflow for Wang et al. DNA methylation changes facilitated evolution of genes derived from *Mutator*-like transposable elements**

1. **Identification of MULE-elements (The following procedure was applied to each of 11 genomes respectively, and we take *O. sativa* ssp. *japonica* genome as a example here):**

RepeatScout-1/build_lmer_table -l 14 -sequence MULE_gene/data/O.sativa.japonica_IRGSP_MSU_v7.fasta -freq MULE_gene/data/Ojapo_repeatscout_freq

RepeatScout-1/RepeatScout -sequence MULE_gene/data/O.sativa.japonica_IRGSP_MSU_v7.fasta -output MULE_gene/data/Ojapo_repeatscout_rm -freq MULE_gene/data/Ojapo_repeatscout_freq -l 14

RepeatMasker/RepeatMasker -pa 20 -s -lib MULE_gene/data/Ojapo_repeatscout_rm_filtered1.fa MULE_gene/data/O.sativa.japonica_IRGSP_MSU_v7.fasta

cat MULE_gene/data/Ojapo_repeatscout_rm_filtered1.fa | RepeatScout-1/filter-stage-2.prl --cat MULE_gene/data/O.sativa.japonica_IRGSP_MSU_v7.fasta.out --thresh 20 > MULE_gene/data/Ojapo_repeatscout_rm_filtered2_20.fa

RepeatMasker/RepeatMasker -pa 20 -s -lib MULE_gene/data/PReDa_RE13_130712.fasta -norna -no_is -gff -cutoff 250 MULE_gene/data/Ojapo_repeatscout_rm_filtered2_20.fa

####PReDa_RE13_130712 was generated by AGI from https://de.iplantcollaborative.org/de/?type=data&folder=/iplant/home/shared/agi_data/Repeat_Annotation/genomes .

cat MULE_gene/data/potential_jiang_seq_newTIR MULE_gene/data/potential_MULE_repeatscout_japo_seq_unredudant > MULE_gene/data/potential_MULE_repeatscout_japo_20_jiang

####potential_jiang_seq_newTIR was downloaded from Ferguson et al. 2013 plant physiology, and potential_MULE_repeatscout_japo_seq_unredudant was generated from Ojapo_repeatscout_rm_filtered2_20.fa after remove the known non-MULE elements and the redundant TE elements.

RepeatMasker/RepeatMasker -pa 20 -s -lib MULE_gene/data/potential_MULE_repeatscout_japo_20_jiang -div 40 -nolow -norna -no_is -gff -cutoff 250 MULE_gene/data/O.sativa.japonica_IRGSP_MSU_v7.fasta_new2

output: MULE_gene/data/O.sativa.japonica_IRGSP_MSU_v7.fasta_new2.out.gff

perl MULE_element/1remove_redudant_rp0.pl

output: MULE_gene/data/O.sativa.japonica_IRGSP_MSU_v7.fasta_new2.out.gff_screen

perl MULE_element/2MULE_scan.pl

output:MULE_gene/data/pack_MULE_candidate_japo_jiang_repeatscout_new_tir_final3

perl MULE_element/3check_num.pl

output:MULE_gene/data/pack_MULE_candidate_japo_jiang_repeatscout_new_tir_final3_name

MULE_gene/data/pack_MULE_candidate_japo_jiang_repeatscout_new_tir_final3_name_5

perl MULE_element/4generate_potential_MULE_denov.pl

MULE_gene/data/potential_MULE_repeatscout_japo_20_jiang_de_nov_candidate

cat MULE_gene/data/potential_jiang_seq_newTIR MULE_gene/data/potential_MULE_repeatscout_japo_20_jiang_de_nov_candidate > MULE_gene/data/potential_MULE_repeatscout_japo_denov_jiang

perl MULE_element/5generate_potential_MULE_non_TIR.pl

MULE_gene/data/non_TIR_MULE.txt_sort_rm_other

cat MULE_gene/data/non_TIR_MULE.txt_sort_rm_other MULE_gene/data/potential_MULE_repeatscout_japo_denov_jiang2 > MULE_gene/data/potential_MULE_repeatscout_japo_denov_jiang_noTIR

RepeatMasker/RepeatMasker -pa 20 -s -lib MULE_gene/data/potential_MULE_repeatscout_glab_denov_jiang_noTIR -div 40 -nolow -norna -no_is -gff -cutoff 250 MULE_gene/data/O.glaberrima.v1.fasta_new3

perl MULE_element/1remove_redudant_rp.pl

output: MULE_gene/data/O.sativa.japonica_IRGSP_MSU_v7.fasta_new3.out.gff_screen

perl MULE_element/6MULE_scan_final.pl

output: MULE_gene/data/pack_MULE_candidate_japo_jiang_repeatscout_new_tir_final5

perl MULE_element/7mule_internal_seq.pl

output:MULE_gene/data/pack_MULE_candidate_japo_jiang_repeatscout_new_tir_final5_internal_seq

./blast-2.2.20/bin/blastall -p tblastn -d MULE_gene/data/pack_MULE_candidate_japo_jiang_repeatscout_new_tir_final5_new_internal_seq -i MULE_gene/data/TE_prot_130507.fasta -o MULE_gene/data/transposase_MULE_japo_new_tir_internal_tblastn_new -m 8

perl MULE_element/8remove_transposase.pl

output:MULE_gene/data/pack_MULE_candidate_japo_jiang_repeatscout_new_tir_final5_new_no_transposase_e9

./blast-2.2.20/bin/blastall -p tblastn -d MULE_gene/data/pack_MULE_candidate_japo_jiang_repeatscout_new_tir_final5_new_internal_seq -i MULE_gene/data/Mutator_transposase.fasta -o MULE_gene/data/auto_MULE_japo_new_tir_internal_tblastn_new -m 8

./blast-2.2.20/bin/blastall -p tblastn -d MULE_gene/data/pack_MULE_candidate_japo_jiang_repeatscout_new_tir_final5_new_no_transposase_e9_internal_seq_only_screen -i MULE_gene/data/maker_protein/oryza_sativa_japonica.protein.fasta -o MULE_gene/data/maker_MULE_japo_new_tir_internal_tblastn_only_new -m 8

perl MULE_element/9remove_auto_MULE.pl

output:MULE_gene/data/pack_MULE_candidate_japo_jiang_repeatscout_new_tir_final5_new_no_transposase_e9_auto_mule

MULE_gene/data/pack_MULE_candidate_japo_jiang_repeatscout_new_tir_final5_new_no_transposase_e9_pack_mule_only

**2. Identification of putative MULE-derived genes**

perl MULE_gene/1Glimmer_annotate_gene.pl

output:MULE_gene/data/pack_MULE_candidate_japo_jiang_repeatscout_new_tir_final5_new_no_transposase_e9_Glimmer

perl MULE_gene/2maker_gene_annotation.pl

output:MULE_gene/data/pack_MULE_candidate_japo_jiang_repeatscout_new_tir_final5_new_no_transposase_e9_coor _involved_exon30

perl MULE_gene/3MULE_gene_annotation.pl

output:MULE_gene/data/pack_MULE_candidate_japo_jiang_repeatscout_new_tir_final5_new_no_transposase_e9_coor_involved_exon30_gff_exon

MULE_gene/data/pack_MULE_candidate_japo_jiang_repeatscout_new_tir_final5_new_no_transposase_e9_coor_involved_exon30_gff_CDS

MULE_gene/data/pack_MULE_candidate_japo_jiang_repeatscout_new_tir_final5_new_no_transposase_e9_coor_involved_exon30_CDS_seq

perl MULE_gene/4translate_pep.pl

output:MULE_gene/data/pack_MULE_candidate_japo_jiang_repeatscout_new_tir_final5_new_no_transposase_e9_coor_involved_exon30_CDS_seq_pep_screen

perl MULE_gene/5annotate_genic_MULE.pl

output:MULE_gene/data/pack_MULE_candidate_japo_jiang_repeatscout_new_tir_final5_new_no_transposase_e9_coor_involved_exon30_gff_exon_genic

MULE_gene/data/pack_MULE_candidate_japo_jiang_repeatscout_new_tir_final5_new_no_transposase_e9_coor_involved_exon30_gff_CDS_genic

MULE_gene/data/pack_MULE_candidate_japo_jiang_repeatscout_new_tir_final5_new_no_transposase_e9_coor_involved_exon30_gff_exon_genic_mule

MULE_gene/data/pack_MULE_candidate_japo_jiang_repeatscout_new_tir_final5_new_no_transposase_e9_coor_involved_exon30_gff_CDS_genic_mule

perl MULE_gene/6MULE_gene_seq.pl

output: MULE_gene/data/pack_MULE_candidate_perr_jiang_repeatscout_new_tir_final5_new_no_transposase_e9_coor_involved_exon30_gff_CDS_genic_gene_seq

perl MULE_gene/7shorten_MULE_gene_name.pl

output:MULE_gene/data/pack_MULE_candidate_japo_jiang_repeatscout_new_tir_final5_new_no_transposase_e9_coor_involved_exon30_gff_CDS_genic_gene_seq_list

MULE_gene/data/pack_MULE_candidate_japo_jiang_repeatscout_new_tir_final5_new_no_transposase_e9_coor_involved_exon30_gff_CDS_genic_gene_seq_short

perl MULE_gene/8MULE_gene_MULE_part.pl

output:MULE_gene/data/pack_MULE_candidate_japo_jiang_repeatscout_new_tir_final5_new_no_transposase_e9_coor_involved_exon30_gff_CDS_genic_gene_seq_list_mule_par

perl MULE_gene/9MULE_gene_MULE_part_seq.pl

output: MULE_gene/data/pack_MULE_japo_gene_mule_part_exon30

RepeatMasker/RepeatMasker -pa 20 -s -lib MULE_gene/data/PReDa_RE13_130712.fasta_repeatscout_rm_mule_japo_new_other2 -norna -no_is -nolow -gff -cutoff 250 MULE_gene/data/pack_MULE_japo_gene_mule_part_exon30

**####Search for the parental sequences of the MULE derived parts of putative MULE-genes and identify the parental genes**

./blast-2.2.20/bin/blastall -p blastn -d MULE_gene/data/O.sativa.japonica_IRGSP_MSU_v7.fasta -i MULE_gene/data/pack_MULE_japo_gene_mule_part_exon30.masked -o MULE_gene/data/japo_MULE_parental_seq_genome_blastn_gene_new1_exon30 -m 8

perl MULE_gene/10MULE_gene_MULE_part_parental.pl

output: MULE_gene/data/japo_MULE_parental_seq_genome_blastn_gene_new1_exon30_parental

perl MULE_gene/11MULE_gene_parental_structure.pl

output: MULE_gene/data/japo_MULE_parental_seq_genome_blastn_gene_new1_exon30_parental_gene_structure

perl MULE_gene/12summarize_parental_gene.pl

output: MULE_gene/data/japo_MULE_parental_seq_genome_blastn_gene_new1_exon30_parental_gene_structure_parental_gene_num

**####Summarize exon number of putative MULE-genes**

perl MULE_gene/13summary_exon_num.pl

output: MULE_gene/data/pack_MULE_candidate_japo_jiang_repeatscout_new_tir_final5_new_no_transposase_e9_coor_involved_exon30_CDS_seq_pep_screen_exon_num

**####Identify non-TE genes**

perl MULE_gene/14no_TE_gene.pl

output: MULE_gene/data/maker_gff1/oryza_sativa_japonica.maker.gff_non_TE_deep

./blast-2.2.20/bin/blastall -p blastp -d MULE_gene/data/maker_protein/oryza_sativa_japonica.protein.fasta -i MULE_gene/data/TE_prot_130507.fasta -o MULE_gene/data/transposase_MULE_japo_protein_new_blastp -m 8

./blast-2.2.20/bin/blastall -p blastp -d MULE_gene/data/maker_protein/oryza_sativa_japonica.protein.fasta -i MULE_gene/data/Mutator_transposase.fasta -o MULE_gene/data/Mu_transposase_MULE_japo_protein_new_blastp -m 8

perl MULE_gene/15screen_nonTE_transposase.pl

output: MULE_gene/data/maker_gff1/oryza_sativa_japonica.maker.gff_non_TE_deep_no_transposase

perl MULE_gene/16screen_nonTE_transposase.pl

output:MULE_gene/data/maker_gff1/oryza_sativa_japonica.maker.gff_non_TE_deep_no_transposase _non_TE_internal

**####Compute GC content of putative MULE-genes, their parental sequences and non-TE genes**

perl MULE_gene/17GC_content.pl

output: japo_MULE_parental_seq_genome_blastn_gene_new1_exon30_parental_parental_seq_gc

pack_MULE_candidate_japo_jiang_repeatscout_new_tir_final5_new_no_transposase_e9_coor_involved_exon30_gff_CDS_genic_gene_seq_gc

pack_MULE_japo_gene_mule_part_exon30_gc

oryza_sativa_japonica.maker.gff_non_TE_deep_no_transposase_non_TE_internal_gene_seq_gc

**####Compute the ka/ks ratio of putative MULE-genes and their paralogs**

perl MULE_gene/18MULE_gene_CDS_seq.pl

output: MULE_gene/data/japo_MULE_gene_all_gene_seq

bin/x86_64-redhat-linux-gnu/blat MULE_gene/data/japo_MULE_internal_seq MULE_gene/data/japo_MULE_gene_all_gene_seq -out=psl -minMatch=0 -minScore=0 -minIdentity=0 MULE_gene/data/japo_MULE_all_MULE.psl

perl MULE_gene/19sort_blat_psl_best.pl

output: MULE_gene/data/japo_MULE_all_MULE.psl_best_sort

perl MULE_gene/20prepare_compute_kaks.pl

output:

MULE_gene/data/japo_MULE_gene_all_para_seq.fa

MULE_gene/data/japo_MULE_gene_all_mule_cds.fa

MULE_gene/data/japo_MULE_gene_all_cds_list

perl MULE_gene/21compute_kaks.pl

output: MULE_gene/data/japo_MULE_all_kaks

MULE_gene/22modified_gKaKs.pl

**3. Phylogeny of MULE elements and putative MULE-genes**

perl Phylogeny/1extract_MULE_and_flanking_seq.pl

output:MULE_gene/data/pack_MULE_candidate_japo_jiang_repeatscout_new_tir_final5_new_no_transposase_e9_flank_seq

output:MULE_gene/data/pack_MULE_candidate_japo_jiang_repeatscout_new_tir_final5_new_no_transposase_e9_flank_list

####Blat japo MULE and flanking sequences against the rest 10 genome sequences.

bin/x86_64-redhat-linux-gnu/blat MULE_gene/data/Lperr_v1.4.fasta MULE_gene/data/pack_MULE_candidate_japo_jiang_repeatscout_new_tir_final5_new_no_transposase_e9_flank_seq -minIdentity=0 MULE_gene/data/MULE_japo_flank_genome_perr.psl

perl Phylogeny/2MULE_ortholog_10_genome.pl

output: MULE_gene/data/pack_MULE_candidate_japo_jiang_repeatscout_new_tir_final5_new_no_transposase_e9_flank_list_niva_ortho

perl Phylogeny/3organize_MULE_ortholog.pl

output: MULE_gene/data/pack_MULE_candidate_japo_jiang_repeatscout_new_tir_final5_new_no_transposase_e9_11_spe_ortho

awk '{split($2,a,":");split($3,b,":");split($4,c,":");if(((a[2]+b[2]+c[2])>=0)&&($5~/0/)&&($6~/0/)&&($7~/0/)&&($8~/0/)&&($9~/0/)&&($10~/0/)&&($11~/0/)){print}}' MULE_gene/data/pack_MULE_candidate_japo_jiang_repeatscout_new_tir_final5_new_no_transposase_e9_11_spe_ortho > MULE_gene/data/pack_MULE_candidate_japo_jiang_repeatscout_new_tir_final5_new_no_transposase_e9_11_spe_ortho_Asian_all

awk '{split($2,a,":");split($3,b,":");split($4,c,":");if(((a[2]+b[2]+c[2])==3)&&($5~/1/)&&($6~/1/)&&($7~/1/)&&($8~/1/)&&($9~/0/)&&($10~/0/)&&($11~/0/)){print}}' MULE_gene/data/pack_MULE_candidate_japo_jiang_repeatscout_new_tir_final5_new_no_transposase_e9_11_spe_ortho > MULE_gene/data/pack_MULE_candidate_japo_jiang_repeatscout_new_tir_final5_new_no_transposase_e9_11_spe_ortho_AA

awk '{split($2,a,":");split($3,b,":");split($4,c,":");split($5,d,":");split($6,e,":");split($7,f,":");split($8,g,":");split($9,h,":"); if(((a[2]+b[2]+c[2]+d[2]+e[2]+f[2]+g[2])>=4)&&(h[2]==1)){print}}' MULE_gene/data/pack_MULE_candidate_japo_jiang_repeatscout_new_tir_final5_new_no_transposase_e9_11_spe_ortho > MULE_gene/data/pack_MULE_candidate_japo_jiang_repeatscout_new_tir_final5_new_no_transposase_e9_11_spe_ortho_BB_all

awk '{if(($2~/0/)&&($3~/0/)&&($4~/0/)&&($5~/0/)&&($6~/0/)&&($7~/0/)&&($8~/0/)&&($9~/0/)&&($10~/0/)&&($11~/0/)){print}}' MULE_gene/data/pack_MULE_candidate_japo_jiang_repeatscout_new_tir_final5_new_no_transposase_e9_11_spe_ortho > MULE_gene/data/pack_MULE_candidate_japo_jiang_repeatscout_new_tir_final5_new_no_transposase_e9_11_spe_ortho_lineage

**####Identify lineage specific putative MULE-genes**

perl Phylogeny/4species_specific_MULE_gene_pep.pl

output: MULE_gene/data/pack_MULE_candidate_japo_jiang_repeatscout_new_tir_final5_new_no_transposase_e9_11_spe_ortho_lineage_gene_list

MULE_gene/data/pack_MULE_candidate_japo_jiang_repeatscout_new_tir_final5_new_no_transposase_e9_11_spe_ortho_lineage_pep_seq

perl Phylogeny/5species_MULE_gene_flank_seq.pl

output: MULE_gene/data/pack_MULE_candidate_japo_jiang_repeatscout_new_tir_final5_new_no_transposase_e9_11_spe_ortho_lineage_gene_list_flank_seq

MULE_gene/data/pack_MULE_candidate_japo_jiang_repeatscout_new_tir_final5_new_no_transposase_e9_11_spe_ortho_lineage_gene_list_flank_list

####Blast japo species-specific MULE-gene peptides against the peptide sequences of the rest 10 genome sequences.

./blast-2.2.20/bin/blastall -p blastp -d MULE_gene/data/maker_protein/oryza_sativa_indica.protein.fasta -i MULE_gene/data/pack_MULE_candidate_japo_jiang_repeatscout_new_tir_final5_new_no_transposase_e9_11_spe_ortho_lineage_pep_seq -o MULE_gene/data/pack_MULE_species_specific_japo_indi_genome_pep2 -m 8

perl Phylogeny/6parse_species_blast_pep.pl

output: MULE_gene/data/pack_MULE_species_specific_japo_genome_pep_ortho2

####Phylogeny/7MULE_gene_ortholog.pl is called in Phylogeny/7MULE_gene_ortholog_together.pl

perl Phylogeny/7MULE_gene_ortholog_together.pl

output: MULE_gene/data/japo_species_specific_blat_indi_genome2.psl_best

MULE_gene/data/pack_MULE_candidate_japo_jiang_repeatscout_new_tir_final5_new_no_transposase_e9_11_spe_ortho_lineage_gene_list_flank_list_indi_ortho1

perl Phylogeny/8organize_species_ortholog.pl

output: MULE_gene/data/pack_MULE_candidate_japo_jiang_repeatscout_final5_new_no_transposase_e9_11_spe_ortho_species

perl Phylogeny/9final_species_ortholog_analysis.pl

output: MULE_gene/data/pack_MULE_candidate_japo_11_spe_ortho_species_genome_pep2

**####Compute the divergence time of MULEs based on the sequence divergence of MULEs and their paralogous sequences with baseml**

perl Phylogeny/10MULE_internal_seq.pl

output: MULE_gene/data/japo_MULE_internal_seq

bin/x86_64-redhat-linux-gnu/blat MULE_gene/data/japo_MULE_internal_seq MULE_gene/data/japo_MULE_internal_seq -out=psl -minMatch=0 -minScore=0 -minIdentity=0 MULE_gene/data/japo_MULE_all_MULE.psl

perl Phylogeny/11best_sort_blat_psl.pl MULE_gene/data/japo_MULE_all_MULE.psl

output: MULE_gene/data/japo_MULE_all_MULE.psl_best_sort

perl Phylogeny/12prepare_paralogs_baseml.pl

output:

MULE_gene/data/japo_MULE_para_1_list

MULE_gene/data/japo_MULE_para_2_list

MULE_gene/data/japo_MULE_para_4_list

MULE_gene/data/japo_MULE_para_6_list

MULE_gene/data/japo_MULE_para_7_list

MULE_gene/data/japo_MULE_para_8_list

MULE_gene/data/japo_MULE_para_9_list

MULE_gene/data/japo_MULE_para_1_list_dir

MULE_gene/data/japo_MULE_para_2_list_dir

MULE_gene/data/japo_MULE_para_4_list_dir

MULE_gene/data/japo_MULE_para_6_list_dir

MULE_gene/data/japo_MULE_para_7_list_dir

MULE_gene/data/japo_MULE_para_8_list_dir

MULE_gene/data/japo_MULE_para_9_list_dir

perl Phylogeny/13run_baseml.pl

output:

MULE_gene/data/japo_MULE_para_1_list_dist

MULE_gene/data/japo_MULE_para_2_list_dist

MULE_gene/data/japo_MULE_para_4_list_dist

MULE_gene/data/japo_MULE_para_6_list_dist

MULE_gene/data/japo_MULE_para_7_list_dist

MULE_gene/data/japo_MULE_para_8_list_dist

MULE_gene/data/japo_MULE_para_9_list_dist

**4. Construct the Marey’s Map**

perl Mareys_Map/1organized_probe.pl

output: MULE_gene/data/distance/rice_prob_chr_all_new

./blast-2.2.20/bin/blastall -p blastn -d MULE_gene/data/maker_gff/oryza_sativa_japonica.cdna.fasta -i MULE_gene/data/distance/rice_prob_chr_all_new -o MULE_gene/data/distance/japo_prob_genome -m 8

perl Mareys_Map/2organized_probe_blast_result.pl

output: MULE_gene/data/distance/japo_prob_genome_single

perl Mareys_Map/3annotate_genetic_distance.pl

output: MULE_gene/data/distance/japo_prob_id_list

MULE_gene/data/distance/chr$id"."_prob_id_gene

perl Mareys_Map/4screen_probe.pl

output: MULE_gene/data/distance/chr$chr_id"."_prob_id_gene_new_pure

perl Mareys_Map/5annotate_nonredudant_physical_distance.pl

output: MULE_gene/data/distance/chr_all_MareyMap_pure_nore

perl Mareys_Map/6MULE_parental_physical_distance.pl

output: MULE_gene/data/japo_MULE_parental_seq_genome_blastn_gene_new1_exon30_parental_phy_dist_all_new

MULE_gene/data/japo_MULE_parental_seq_genome_blastn_gene_new1_exon30_parental_phy_dist_all_list_new

perl Mareys_Map/7nonTE_gene_physical_distance.pl

output: MULE_gene/data/maker_gff1/oryza_sativa_japonica.maker.gff_non_TE_deep_no_transposase_non_TE_internal_phy_dist_all_new

MULE_gene/data/maker_gff1/oryza_sativa_japonica.maker.gff_non_TE_deep_no_transposase_non_TE_internal_phy_dist_all_list_new

**5. Analysis of methylation pattern of genic-MULE and putative MULE-genes.**

perl methylation/1prepare_coordinate_nonTE_gene.pl

output: MULE_gene/data/japo_internal_MULE_c_coor_gene_no_te_maker_new1_mid200_deep_random_gene_nonTE

perl methylation/2prepare_coordinate_parental.pl

output: MULE_gene/data/japo_internal_MULE_c_coor_par_new_masked_gene_mule_parental_seq_new1

perl methylation/3prepare_coordinate_MULE.pl

output: MULE_gene/data/japo_internal_MULE_c_coor_single_new

perl methylation/4prepare_coordinate_MULE_gene.pl

output: MULE_gene/data/japo_internal_MULE_c_coor_gene_new

perl methylation/5prepare_coordinate_MULE_promoter.pl

output: MULE_gene/data/japo_internal_MULE_c_coor_promoter_new

perl methylation/6prepare_coordinate_MULE_flanking.pl

output: MULE_gene/data/japo_internal_MULE_c_coor_single_tir_fl

perl methylation/7prepare_coordinate_MULE_TIR.pl

output: MULE_gene/data/japo_internal_MULE_c_coor_single_tir

perl methylation/8computate_methylation_level.pl MULE_gene/data/japo_internal_MULE_c_coor_par_new_masked_gene_mule_parental_seq_new1 _japo_methy_w

output:

MULE_gene/data/japo_internal_MULE_c_coor_par_new_masked_gene_mule_parental_seq_new1_japo_methy_w_methy3_1

MULE_gene/data/japo_internal_MULE_c_coor_par_new_masked_gene_mule_parental_seq_new1_japo_methy_w_methy3_1_stat

perl methylation/9screen_genic_non_genic_MULE.pl

output: MULE_gene/data/japo_internal_MULE_c_coor_gene_new_japo_methy_w_methy3_1_stat_genic1

MULE_gene/data/japo_internal_MULE_c_coor_gene_new_japo_methy_w_methy3_1_stat_nongenic1

perl methylation/10screen_out_auto_mule_4stage.pl

output: MULE_gene/data/japo_internal_MULE_c_coor_single_new_japo_methy_w_methy3_1_stat_genic1_genic_non_auto_lineage_new

MULE_gene/data/japo_internal_MULE_c_coor_single_new_japo_methy_w_methy3_1_stat_genic1_genic_non_auto_Asian_all_new

MULE_gene/data/japo_internal_MULE_c_coor_single_new_japo_methy_w_methy3_1_stat_genic1_genic_non_auto_AA_new

MULE_gene/data/japo_internal_MULE_c_coor_single_new_japo_methy_w_methy3_1_stat_genic1_genic_non_auto_BB_all_new

perl methylation/11sliding_window_analysis.pl MULE_gene/data/japo_internal_MULE_c_coor_single_new_japo_methy_w_methy3_1_stat_genic1_genic_non_auto_BB_all_new temp_file3 temp_err3

output: MULE_gene/data/methy_slide_wd_f2/ japo_internal_MULE_c_coor_single_new_japo_methy_w_methy3_1_stat_genic1_genic_non_auto_BB_all_new

**####Compute TE coverage**

RepeatMasker/RepeatMasker -pa 20 -s -lib MULE_gene/data/PReDa_RE13_130712.fasta_repeatscout_rm_mule_japo_new_other2 -norna -no_is -nolow -gff -cutoff 250 MULE_gene/data/pack_MULE_candidate_japo_jiang_repeatscout_new_tir_final5_new_no_transposase_e9_internal_seq_only_screen

perl methylation/12TE_coverage.pl

output: MULE_gene/data/pack_MULE_candidate_japo_jiang_repeatscout_new_tir_final5_new_no_transposase_e9_internal_seq_only_screen.out.gff_cover

perl methylation/13screen_out_auto_mule_4stage_TE_coverage.pl

output: MULE_gene/data/pack_MULE_candidate_japo_jiang_repeatscout_new_tir_final5_new_no_transposase_e9_coor_involved_exon30_gff_CDS_genic_mule_ genic_non_auto_Asian_all_te_cov

MULE_gene/data/pack_MULE_candidate_japo_jiang_repeatscout_new_tir_final5_new_no_transposase_e9_coor_involved_exon30_gff_CDS_genic_mule_ genic_non_auto_AA_te_cov

MULE_gene/data/pack_MULE_candidate_japo_jiang_repeatscout_new_tir_final5_new_no_transposase_e9_coor_involved_exon30_gff_CDS_genic_mule_ genic_non_auto_BB_all_te_cov

#####compute the identity of TIR sequences

perl methylation/14compute_TIR_identity.pl

output:MULE_gene/data/pack_MULE_candidate_japo_jiang_repeatscout_new_tir_final5_new_no_transposase_e9_ tir_mafft_result

Perl methylation/15separate_tir_identity_4stage.pl

output: MULE_gene/data/pack_MULE_candidate_japo_jiang_repeatscout_new_tir_final5_new_no_transposase_e9_tir_mafft_result_Asian_all_genic_new

MULE_gene/data/pack_MULE_candidate_japo_jiang_repeatscout_new_tir_final5_new_no_transposase_e9_tir_mafft_result_AA_genic_new

MULE_gene/data/pack_MULE_candidate_japo_jiang_repeatscout_new_tir_final5_new_no_transposase_e9_tir_mafft_result_BB_all_genic_new

**6. Analysis of small RNA pattern of genic MULEs and pollen expression of putative MULE-genes**

**####Analyze small RNA pattern of genic-MULEs**

/wsu/arch/x86_64/genomics/bwa/bwa-12-17-2013-git/bwa samse -n 99 MULE_gene/data/O.sativa.japonica_IRGSP_MSU_v7.fasta MULE_gene/data/sRNA_tophat_BCP/BCP_bwa.sai MULE_gene/data/sRNA/BCP_processed.txt_fa > MULE_gene/data/sRNA_tophat_BCP/BCP.bwa.sam

perl sRNA_pollen_expression/1extract_uniq_mapping_sRNA.pl

output: MULE_gene/data/sRNA_tophat_TCP/TCP_sort_sRNA_list_uniq_bwa0

MULE_gene/data/sRNA_tophat_TCP/TCP_sort_sRNA_list_two_more_bwa0

perl sRNA_pollen_expression/2count_sRNA_in_MULE.pl

output: MULE_gene/data/sRNA_tophat_TCP/TCP_sort_sRNA_list_uniq_bwa0_sort_mule_clean

MULE_gene/data/sRNA_tophat_TCP/TCP_sort_sRNA_list_two_more_bwa0_sort_mule_clean

perl sRNA_pollen_expression/3count_sRNA_in_TIR.pl

output: MULE_gene/data/sRNA_tophat_TCP/TCP_sort_sRNA_list_uniq_bwa0_sort_tir_clean

MULE_gene/data/sRNA_tophat_TCP/TCP_sort_sRNA_list_two_more_bwa0_sort_tir_clean

awk '{if($5>=0.5){print}}' MULE_gene/data/japo_internal_MULE_c_coor_single_new_japo_methy_w_methy3_1_stat_genic1_genic_non_auto_Asian_all_new | grep "CG" | cat > MULE_gene/data/japo_internal_MULE_c_coor_single_new_methy_stat_w_genic_Asian_all_new

awk '{if($5>=0.5){print}}' MULE_gene/data/japo_internal_MULE_c_coor_single_new_japo_methy_w_methy3_1_stat_genic1_genic_non_auto_AA_new | grep "CG" | cat > MULE_gene/data/japo_internal_MULE_c_coor_single_new_methy_stat_w_genic_AA_new

awk '{if($5>=0.5){print}}' MULE_gene/data/japo_internal_MULE_c_coor_single_new_japo_methy_w_methy3_1_stat_genic1_genic_non_auto_BB_all_new | grep "CG" | cat > MULE_gene/data/japo_internal_MULE_c_coor_single_new_methy_stat_w_genic_BB_all_new

perl sRNA_pollen_expression/4count_sRNA_MULE_stage.pl

output:

MULE_gene/data/japo_internal_MULE_c_coor_single_new_methy_stat_w_genic_Asian_all_new_sRNA_all_uniq_bwa0_mutant_mule_mutant_new

MULE_gene/data/japo_internal_MULE_c_coor_single_new_methy_stat_w_genic_BB_all_new_sRNA_all_uniq_bwa0_mutant_mule_mutant_new

MULE_gene/data/japo_internal_MULE_c_coor_single_new_methy_stat_w_genic_AA_new_sRNA_all_uniq_bwa0_mutant_mule_mutant_new

MULE_gene/data/japo_internal_MULE_c_coor_single_new_methy_stat_w_genic_Asian_all_new_sRNA_all_two_more_bwa0_mutant_mule_mutant_new

MULE_gene/data/japo_internal_MULE_c_coor_single_new_methy_stat_w_genic_BB_all_new_sRNA_all_two_more_bwa0_mutant_mule_mutant_new

MULE_gene/data/japo_internal_MULE_c_coor_single_new_methy_stat_w_genic_AA_new_sRNA_all_two_more_bwa0_mutant_mule_mutant_new

perl sRNA_pollen_expression/5count_sRNA_TIR_stage.pl

output: MULE_gene/data/japo_internal_MULE_c_coor_single_new_methy_stat_w_genic_Asian_all_new_sRNA_all_uniq_bwa0_mutant_tir_mutant_new

MULE_gene/data/japo_internal_MULE_c_coor_single_new_methy_stat_w_genic_AA_new_sRNA_all_uniq_bwa0_mutant_tir_mutant_new

MULE_gene/data/japo_internal_MULE_c_coor_single_new_methy_stat_w_genic_BB_all_new_sRNA_all_uniq_bwa0_mutant_tir_mutant_new

MULE_gene/data/japo_internal_MULE_c_coor_single_new_methy_stat_w_genic_Asian_all_new_sRNA_all_two_more_bwa0_mutant_tir_mutant_new

MULE_gene/data/japo_internal_MULE_c_coor_single_new_methy_stat_w_genic_AA_new_sRNA_all_two_more_bwa0_mutant_tir_mutant_new

MULE_gene/data/japo_internal_MULE_c_coor_single_new_methy_stat_w_genic_BB_all_new_sRNA_all_two_more_bwa0_mutant_tir_mutant_new

perl sRNA_pollen_expression/6summarize_sRNA_MULE_and_TIR.pl

output:

MULE_gene/data/japo_4_pack_mule_11_tissue_sRNA_species_test_all_japo_methy_w_uniq

**####Analyze pollen expression data**

~/sratoolkit.2.1.9-centos_linux64/fastq-dump -SL --split-3 -A MULE_gene/data/RNA_seq/SRR074144.sra

./trim_galore -q 20 -a TCGTA MULE_gene/data/RNA_seq/MULE_gene_data_RNA_seq_SRR074144.sra.fastq --length 15 -o MULE_gene/data/RNA_seq/

tophat-2.0.10.Linux_x86_64/tophat -o MULE_gene/data/RNA_seq/SRR074144 -p 10 MULE_gene/data/japo_index/ MULE_gene/data/RNA_seq/MULE_gene_data_RNA_seq_SRR074144.sra_trimmed.fq

/wsu/apps/pre-compiled/cufflinks/cufflinks-2.2.1/cufflinks -o MULE_gene/data/RNA_seq/SRR074144 -p 15 -b MULE_gene/data/O.sativa.japonica_IRGSP_MSU_v7.fasta -u -G MULE_gene/data/pack_MULE_candidate_japo_jiang_repeatscout_new_tir_final5_new_no_transposase_e9_coor_involved_exon30_gff_exon_genic_cuff MULE_gene/data/RNA_seq/SRR074144/accepted_hits.bam

/wsu/apps/pre-compiled/cufflinks/cufflinks-2.2.1/cufflinks -o MULE_gene/data/RNA_seq/SRR074144_gff -p 15 -b MULE_gene/data/O.sativa.japonica_IRGSP_MSU_v7.fasta -u -G MULE_gene/data/maker_gff1/oryza_sativa_japonica.maker.gff_cuff MULE_gene/data/RNA_seq/SRR074144/accepted_hits.bam

perl sRNA_pollen_expression/7all_tissue_expression_proportion.pl

output: MULE_gene/data/RNA_seq/japo_exp_all_new1

perl sRNA_pollen_expression/8all_tissue_relative_expression.pl

output: MULE_gene/data/RNA_seq/japo_exp_all_norm3

**7. The R commands are available in the R_command folder of https://github.com/FanLabWayneStateU/MULE-methylation**
