# Supplementary material for: DNA methylation changes facilitated evolution of genes derived from Mutator-like transposable elements
Source: Genome Biol. 2016 May 6;17:92. doi: 10.1186/s13059-016-0954-8 (PMC4858842; doi:10.1186/s13059-016-0954-8)
Supplement: Additional file 4: — Supplementary file S2. Accession numbers and URLs for genome assembly, transcriptome and methylome data that used in this project. (DOCX 101 kb) [file 13059_2016_954_MOESM4_ESM.docx]

**Supplementary File S2**. Accession numbers and URLs for genome assembly, transcriptome and methylome data that used in this project.

All genome assembly data are publically available from the National Center for Biotechnology Information (NCBI), and the accession numbers are: *L. perrieri*: ALNV00000000.2; *O. barthii*: ABRL00000000.2; *O. brachyantha*: AGAT00000000.1; *O. glaberrima*: ADWL00000000.1; *O. glumaepatula*: ALNU00000000.2; *O. meridionalis*: ALNW00000000.2; *O. nivara*: AWHD00000000.1; *O. punctata*: AVCL00000000.1; and *O. rufipogon*: PRJEB4137. Whole genome sequences of *O. sativa* ssp. *japonica* were obtained from <http://rice.plantbiology.msu.edu>, and that of *O. sativa* ssp. *indica* was downloaded from <http://rise2.genomics.org.cn/page/rice/download.jsp>.

Transcriptome RNA-seq data are available from NCBI Sequence Read Archive (SRA), and accession numbers are: *O. rufipogon* (SRX512340, SRX512341, and SRX512342), *O. nivara* (SRX472708, SRX472709, and SRX472710), *O. barthii* (SRX471823, SRX472434, and SRX472435), *O. glaberrima* (SRX474528, SRX474529, and SRX474530), *O. sativa* ssp. *japonica* (SRX477950, SRX477951, and SRX477952), *O. glumaepatula* (SRX475002, SRX475003 and SRX475004), *O. brachyantha (*SRX475011*)*, *O. meridionalis* (SRX475006, SRX475007, and SRX475008), *O. punctata* (SRX472098, SRX472099, and SRX472100), and *L. perrieri* (SRX472913, SRX472914, and SRX472915).

Methylome data for *O. sativa* ssp. *japonica* and *O. nivara* used in this project are available from iPlant Collaborative (http://www.iplantcollaborative.org/) data store at <https://de.iplantcollaborative.org/de/?type=data&folder=/iplant/home/shared/agi_data/Rice_methylome_data/genome_matrices/O_sativa_japonica>, and <https://de.iplantcollaborative.org/de/?type=data&folder=/iplant/home/shared/agi_data/Rice_methylome_data/genome_matrices/O_nivara>.
